# Associations of polygenic risk score, environmental factors, and their interactions with the risk of schizophrenia spectrum disorders

**DOI:** 10.1017/S0033291725000753

**Published:** 2025-04-11

**Authors:** Fatima Zahra Rami, Hyungwoo Seo, Chaeyeong Kang, Seunghwan Park, Ling Li, Thi-Hung Le, Sung-Wan Kim, Seung-Hee Won, Wonil Chung, Young-Chul Chung

**Affiliations:** 1 Research Institute of Clinical Medicine of Jeonbuk National University and Biomedical Research Institute of Jeonbuk National University Hospital, Jeonju, South Korea; 2 Department of Statistics and Actuarial Science, Soongsil University, Seoul, South Korea; 3 Department of Psychiatry, Jeonbuk National University Medical School, Jeonju, South Korea; 4 Department of Psychiatry, Chonnam National University Medical School, Gwangju, South Korea; 5 Department of Psychiatry, School of Medicine, Kyungpook National University, Daegu, South Korea; 6 Program in Genetic Epidemiology and Statistical Genetics, Harvard T.H. Chan School of Public Health, Boston, MA, USA

**Keywords:** gene–environment interactions, Korea-Polyenvironmental Risk Score, polygenic risk score, Schizophrenia spectrum disorders

## Abstract

**Background:**

Emerging evidence indicates that gene–environment interactions (GEIs) are important underlying mechanisms for the development of schizophrenia (SZ). We investigated the associations of polygenic risk score for SZ (PRS-SZ), environmental measures, and their interactions with case–control status and clinical phenotypes among patients with schizophrenia spectrum disorders (SSDs).

**Methods:**

The PRS-SZ for 717 SSD patients and 356 healthy controls (HCs) were calculated using the LDpred model. The Korea-Polyenvironmental Risk Score-I (K-PERS-I) and Early Trauma Inventory-Self Report (ETI-SR) were utilized as environmental measures. Logistic and linear regression analyses were performed to identify the associations of PRS-SZ and two environmental measures with case–control status and clinical phenotypes.

**Results:**

The PRS-SZ explained 8.7% of SZ risk. We found greater associations of PRS-SZ and total scores of the K-PERS-I with case–control status compared to the ETI-SR total score. A significant additive interaction was found between PRS-SZ and K-PERS-I. With the subdomains of the K-PERS-I and ETI-SR, we identified significant multiplicative or additive interactions of PRS-SZ and parental socioeconomic status (pSES), childhood adversity, and recent life events in association with case–control status. For clinical phenotypes, significant interactions were observed between PRS-SZ and the ETI-SR total score for negative-self and between PRS-SZ and obstetric complications within the K-PERS-I for negative-others.

**Conclusions:**

Our findings suggest that the use of aggregate scores for genetic and environmental measures, PRS-SZ and K-PERS-I, can more accurately predict case–control status, and specific environmental measures may be more suitable for the exploration of GEIs.

## Introduction

Schizophrenia (SZ) is a complex disorder influenced by various factors, including genetic and environmental factors. Genetic factors explain a substantial portion of the risk, as indicated by twin studies demonstrating >80% heritability (Sullivan, Kendler, & Neale, 2003). However, environmental factors, particularly a history of significant childhood adversity (CA), also play key roles in the development and course of the disease (Murray et al., 2020). The relationships among these factors have been extensively discussed, and hypotheses of gene–environment interactions (GEIs) for SZ have been proposed (Murray, Reveley, & McGuffin, 1986; Schulsinger, Parnas, Mednick, Teasdale, & Schulsinger, 1987; Strahilevitz, 1974). The identification of GEI effects in psychiatric disorders offers three key benefits: it allows new genetic and environmental main effects to be discovered (Kraft, Yen, Stram, Morrison, & Gauderman, 2007), it enhances the understanding of the underlying biological pathways (Caspi & Moffitt, 2006), and it offers clinical insights for personalized medicine and lifestyle recommendations (Dempfle et al., 2008; Uher & McGuffin, 2008). Although GEIs are important, their identification requires sample sizes ranging from thousands (for candidate genes) to tens of thousands (for genome-wide association studies); thus, the statistical power can be limited (Thomas, 2010).

When used as a single metric of molecular genetic risk, the polygenic risk score (PRS) has greatly enhanced the capacity for identifying associations with phenotypes and GEIs (Choi, Mak, & O’Reilly, 2020; Lin, Huang, Liu, Tsai, & Kuo, 2019). There is evidence that substantially greater predictive power can be achieved using the PRS rather than a small number of genome-wide significant single nucleotide polymorphisms (SNPs) (Domingue, Trejo, Armstrong-Carter, & Tucker-Drob, 2020; Dudbridge, 2013). Many recent psychiatry studies have utilized PRS approaches to investigate GEIs (Soga, Teo, & Parhar, 2021; Woolway et al., 2022; Yao, van der Veen, Thygesen, Bass, & McQuillin, 2023). In SZ, prior studies have reported positive interactions between the PRS for SZ (PRS-SZ) and factors such as cannabis use (Gage et al., 2017; Guloksuz et al., 2019; Pasman et al., 2018), urbanicity (Colodro-Conde et al., 2018; Maxwell, Coleman, Breen, & Vassos, 2021; Paksarian et al., 2018), and CA (Guloksuz et al., 2019; Mas-Bermejo et al., 2023; Pignon et al., 2022; Pries et al., 2020a; Pries et al., 2020b; Saarinen et al., 2024; Sallis et al., 2021; Smigielski et al., 2021; Trotta et al., 2016). However, contrasting findings also exist; some authors have reported negative associations between PRS-SZ and cannabis use (Johnson et al., 2023) and CA (Guloksuz et al., 2019; Mas-Bermejo et al., 2023; Pignon et al., 2022; Pries, Klingenberg, et al., 2020a; Pries, Klingenberg, et al., 2020b; Saarinen et al., 2024; Sallis et al., 2021; Smigielski et al., 2021; Trotta et al., 2016).

Although most studies have primarily focused on GEIs using single environmental factors, each factor represents a small portion of the dense network of possible environmental exposures. The introduction of a cumulative environmental score would provide a single measure, thereby enhancing risk prediction and advancing research to more fully comprehend the collective impact of the environment and its interactions with genetics in the context of psychosis. Several tools have been developed to measure cumulative environmental load in the form of a single aggregate score, similar to the PRS used in genetics. Examples include the exposome score for SZ (ES-SZ) (Cuesta et al., 2023; Pries et al., 2020), the Maudsley Environmental Risk Score (ERS) (Vassos et al., 2020), the Polyenviromic Risk Score (PERS) (Padmanabhan, Shah, Tandon, & Keshavan, 2017), and the Psychosis Polyrisk Score (PPS) (Oliver et al., 2020; Oliver, Radua, Reichenberg, Uher, & Fusar-Poli, 2019). While these tools incorporate similar risk factors, they differ in how the aggregate score is calculated. ERS and PPS scores are estimated by scaling odds ratios (ORs) or relative risks (RRs) with population prevalence for each risk factor, and the PERS score is obtained by simply summing the ORs of the risk factors. ES-SZ employs a more advanced approach using weighted coefficients derived from a single predictive model, which accounts for the interdependency of exposures (Pries et al., 2019). In contrast, the K-PERS-I score is calculated using ORs and RRs from Western studies, adjusted for the population proportions of risk factors identified from multiple resources of the Korean data. However, only a limited number of studies have investigated GEIs using these tools in SZ, and they have yielded mixed results (Cuesta et al., 2023; Guloksuz et al., 2019; Mas et al., 2020; Pries, Dal Ferro, et al., 2020). Notably, there has been a lack of studies examining GEIs in Asian SZ patients using these comprehensive measures of polyenvironmental factors.

We utilized the Korea-Polyenvironmental Risk Score-I (K-PERS-I) (Jeon et al., 2022), a comprehensive measure of multiple environmental factors associated with SZ, developed based on the proportions of risk factors (exposure) in the Korean population. Moreover, considering suggestions that GEIs can only involve monogenic factors (Caspi et al., 2005; Stefanis et al., 2007) and comprehensive measures may not be appropriate tools for studying GEIs (Assary, Vincent, Keers, & Pluess, 2018), we also adopted three specific environmental measures: subdomains of the K-PERS-I, as well as the total and subdomains of the Early Trauma Inventory-Self Report (ETI-SR) (Bremner, Bolus, & Mayer, 2007), which evaluates CA.

In this study, we aimed to calculate PRS-SZ using the Korean Genomics Center (KOGIC) dataset and East Asia (EAS) summary statistics from the Psychiatric Genomics Consortium (PGC). Subsequently, using the two aggregate scores (PRS-SZ and total K-PERS-I), we investigated the associations of two main factors and their interactions with case–control status, as well as with clinical phenotypes assessed using the Brief Core Schema Scales (BCSS) and the Positive and Negative Syndrome Scale (PANSS) in patients with schizophrenia spectrum disorders (SSDs). Additionally, we explored these associations using three specific environmental measures. The subsequent results were compared in order to see the possible advantages of using the comprehensive measure, K-PERS-I, over three specific environmental measures.

## Methods

### Participants

Patients (*n* = 818) were recruited from outpatients and inpatients treated at four hospitals (Jeonbuk [JB], Chonnam, Kyungpook, and Haeundae Paik National University Hospitals [NUH]) from December 2014 to February 2021. Inclusion criteria were as follows: (a) SSDs including SZ, schizoaffective disorder, schizophreniform disorder, and psychotic disorder not otherwise specified; (b) and age between 18 and 59 years. Diagnoses were established using the DSM-IV-TR criteria (American Psychiatric Association, 1994) and the Korean version of the Mini-International Neuropsychiatric Interview (Yoo et al., 2006). The exclusion criteria were as follows: (a) intelligence quotient (IQ) ≤ 70; (b) acute, unstable, or severe medical/neurological conditions; or (c) pregnancy or lactation. Healthy controls (HCs) were recruited through advertisements at JBNUH (*n* = 356) and underwent psychiatric interviews using the screening module of the Structured Clinical Interview for DSM-IV (First, Spitzer, Gibbon, & Williams, 2002). HCs were excluded if they had a current or previous diagnosis of mental disorders, a clinically significant medical condition, or first-degree relatives with psychosis (to minimize the effects of genetic loading). The authors assert that all procedures contributing to this work complied with the ethical standards of relevant national and institutional Human Experimental Commissions and the 1975 Declaration of Helsinki, as amended in 2008. All procedures involving human subjects/patients were approved by the Ethics Committee of JBNUH (approval number CUH 2014-11-002).

### Assessments

Psychopathology was evaluated using PANSS (Kay, Fiszbein, & Opler, 1987; Yi et al., 2001). To maximize inter-rater reliability across sites, psychiatrists with more than 3 years of experience in this field participated in the ratings process, and several workshops were held during the recruitment period (Cohen’s kappa ≥0.8). Self-rating scales, such as BCSS (Fowler et al., 2006) and ETI-SR, were administered. For simplicity, we used only two subdomains of the ETI-SR, negative-self and negative-others, by reversing the scores of positive-self and positive-others. The reliability and validity of the Korean version of the ETI-SR were confirmed in Korean patients with depression (Cronbach’s alpha = 0.87) (Jeon et al., 2012). Environmental risk factors associated with SZ were measured using the K-PERS-I (Jeon et al., 2022), which consisted of six domains: paternal age at birth, parental socioeconomic status (pSES), obstetric complications, urbanicity, CA, and recent life events. For domain and scoring system definitions, see Supplementary Tables: Material 1.

### Calculation of the PRS-SZ

The JBNUH genotype dataset (for details on the quality control of genetic data, see Supplementary Tables: Material 2 and Supplementary Figures 1 and 2), was converted from UCSC hg19 genomic coordinates to hg38 using CrossMap (v0.6.4). The KOGIC dataset of the Ulsan National Institute of Science and Technology consists of 28,692,913 SNPs in 1,047 Korean individuals, primarily from the Ulsan metropolitan region in the southern part of the Korean peninsula (Jeon et al., 2020). After the conversion, the dataset retained 437,188 SNPs. KOGIC genotype data were used as a reference panel for genotype imputation in the JBNUH dataset, conducted in two stages. First, pre-phasing of each chromosome was performed using Eagle (v2.4.1). Subsequently, Minimac3 (v2.0.1) was used to impute phased genotypes for approximately 28.7 million markers in the KOGIC genotype data, retaining variants with an imputation INFO score > 0.8. A global PRS-SZ was then generated using East Asian (EAS) summary statistics (Lam et al., 2019) and KOGIC data as the linkage disequilibrium (LD) reference panel. For the PRS-SZ calculation, we utilized 2,525,966 SNPs that overlapped with the SNPs in the EAS summary statistics. Instead of limiting the analysis to significant SNPs, we included all available SNPs. The effect sizes were reweighted using the LDpred-inf algorithm to account for causal variants and non-infinitesimal genetic architecture. This approach incorporates a point-normal prior for posterior mean effect size estimation, implemented via the Markov Chain Monte Carlo (MCMC) method, which assumes a Gaussian mixture prior: 
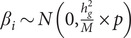
 with probability *p*, and 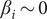 with probability 1 − *p*, where *p* is the proportion of causal SNPs. The method is optimized by considering different values of *p* (10^−3^, 3 × 10^−3^, 0.01, 0.03, 0.1, 0.3, 1) (Privé, Arbel, & Vilhjálmsson, 2021; Vilhjálmsson et al., 2015). The study population *n* = 1,073 participants (717 with SZ) was tested for the predictive performance of the global PRS-SZ. Personal scores were computed by coding each variant, weighting by its relative effect size on SZ, along with standardization. The PRS-SZ with the highest discriminative capability was chosen based on the maximum area under the curve (AUC). The estimated AUCs for different proportions of causal variants (10^−3^, 3 × 10^−3^, 0.01, 0.03, 0.1, 0.3, 1) were 0.529, 0.670, 0.695, 0.700, 0.699, 0.698, and 0.699, respectively. The LDpred model with a 3% proportion of causal variants demonstrated the highest predictive accuracy for SZ. Furthermore, age and sex adjustments were applied to the PRS-SZ. We calculated the heritability 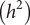 for SZ with the Korean-specific LD scores and EAS summary statistics using LD score regression (Bulik-Sullivan et al., 2015). The prediction accuracy for the PRS-SZ was assessed using AUC, which was subsequently converted to liability *R*^2^, considering a population lifetime risk of SZ at 1% (Lee, Wray, Goddard, & Visscher, 2011).

### Statistical analyses

Gene–environment correlation between PRS-SZ and the subdomains of K-PERS-I in SSDs patients was assessed using a logistic regression model, adjusted for age, sex, and education. To examine the associations of PRS-SZ and/or total scores of K-PERS-I/ETI-SR with case–control status, we conducted four types of logistic regression models: (i) a genetic model with PRS-SZ only, (ii) an environmental model including either K-PERS-I or ETI-SR, (iii) an independent model with PRS-SZ and K-PERS-I/ETI-SR without an interaction term, and (iv) an interaction model with PRS-SZ and K-PERS-I/ETI-SR with an interaction term. For the total score of the K-PERS-I/ETI-SR, we performed analyses using a complete dataset and an imputed dataset. Interaction was assessed in two ways: first, through logistic regression to evaluate interaction as a departure from multiplicativity, and second, through the Relative Excess Risk due to Interaction (RERI) to assess interaction as a departure from additivity (Knol & VanderWeele, 2012). An RERI >0 indicated a positive deviation from additivity and was considered statistically significant if the 95% confidence interval (CI) did not contain zero. The RERIs were calculated using the delta method, which imposes an unnatural symmetry on the confidence limits of the underlying effect measure (Hosmer & Lemeshow, 1992), using the ORs derived from each model via the epiR package in R. In all analyses, we adjusted for covariates such as age, sex, and education. Multiple testing for subdomains of the K-PERS-I was corrected with false discovery rate (FDR). Nagelkerke’s *R*^2^ value for each model was calculated with the DescTools package in R (version 0.99.52).

Additionally, logistic regression analyses were conducted with dichotomized PRS-SZ and K-PERS-I/ETI-SR scores, using a threshold of 75% of the HC group. This threshold was selected to more effectively capture the impact of high genetic risk while minimizing the potential masking of effects by intermediate or low scores (Guloksuz et al., 2019; Mas et al., 2020; Pries, Dal Ferro, et al., 2020; Segura et al., 2023). The subgroup with low K-PERS-I/ETI-SR and low PRS-SZ with scores less than 75% was regarded as the reference group. Sensitivity analyses were performed using alternative thresholds of 50% and 25%. We also conducted the same analysis separately for males and females. Moreover, considering that simply adjusting for these covariates could be insufficient to eliminate potential bias in the Gene × Environment interaction term (Keller, 2014), we performed further analyses with these covariates included as interaction terms and assessed the improvement of model fit with the Akaike Information Criterion (AIC) and the Bayesian Information Criterion (BIC).

To investigate the associations of PRS-SZ and K-PERS-I/ETI-SR (total and subdomains) and their interactions with clinical phenotypes, PANSS, and BCSS, linear regression with the interaction model was utilized. Multiple testing correction was applied to all analyses using the FDR method, with a significance threshold of FDR < 0.05. As we had smaller sample sizes for the total K-PERS-I/ETI-SR because of different missing data in the subdomains of the K-PERS-I/ETI-SR, we conducted the main analyses again after the imputation using the mice package in R (version 3.16.0). In all analyses, we used R software (ver. 4.1.1) and adjusted for age, sex, and education as covariates.

For power calculation, we performed post hoc power calculation (Rodriguez et al., 2024) for the PRS-SZ by K-PERS-I interaction using a simulation method in R using the standardized coefficients for PRS-SZ, K-PERS-I, and PRS-SZ * K-PERS-I, from the comparison of case–control (For details on the code used, see Supplementary Tables: Materials 3 and 4).

## Results

### Demographic and clinical characteristics of the participants

The participants were divided into three groups: SSDs with K-PERS-I (*n* = 295–398), SSDs with ETI-SR (*n* = 474–476), and HCs (*n* = 320–356). Compared with HCs, the SSDs with K-PERS-I group were younger (*p* < 0.001); it had higher negative-self and negative-others scores (*p* < 0.001), and higher total and subdomains K-PERS-I scores (*p* < 0.001), with the exception of pSES. Similarly, compared with the HCs, the SSDs with ETI-SR group were younger (*p* < 0.001); it also had higher negative-self and negative-others scores (*p* < 0.001) and lower total and subdomain ETI-SR scores. Comparison of the SSDs with K-PERS-I and the SSDs with ETI-SR groups revealed no significant differences (Table 1).

**Table 1.** Demographic and clinical characteristics of the participants

	SSDs (*n* = 818)	SSDs with K-PERS-I (*n* = 280 ~ 398)	SSDs with ETI-SR (*n* = 432 ~ 476)	HCs (*n* = 320 ~ 356)	p-value^a^	p-value^b^	p-value^c^
Site, *n* (%)					–		<0.001
Jeonbuk NUH	485 (59.3)	246 (83.4)	358 (75.5)	356 (100)			
Chonnam NUH	317 (38.8)	42 (14.2)	101 (21.3)				
Kyungpook NUH	9 (1.1)	7 (2.4)	9 (1.9)				
Haeundae Paik NUH	7 (0.9)	–	6 (1.3)				
Age	34.3 ± 11.3 (*n* = 818)	36 ± 11.6 (*n* = 295)	35.3 ± 11.5 (*n* = 474)	41.0 ± 14.3 (*n* = 356)	<0.001	<0.001	0.333
Sex, n (%)					0.206	0.726	0.754
Male	386 (47.2)	137 (46.4)	230 (48.5)	178 (50.0)			
Female	432 (52.8)	158 (53.6)	244 (51.5)	178 (50.0)			
Education	13.5 ± 2.6 (*n* = 717)	13.7 ± 2.6 (*n* = 295)	13.6 ± 2.6 (*n* = 474)	13.9 ± 2.4 (*n* = 356)	0.380	0.157	0.304
DI	110.7 ± 119.6 (*n* = 600)	111.7 ± 120.1 (*n* = 292)	108.9 ± 121.9 (*n* = 473)	–			0.591
Diagnosis, n (%)				–			0.546
Schizophrenia	731 (89.4)	249 (84.4)	414 (87.3)				
Schizophreniform	80 (9.8)	44 (14.9)	58 (12.2)				
Schizoaffective	1 (0.1)	–	–				
PNOS	6 (0.7)	2 (0.7)	2 (0.4)	–			
PANSS							
Total	60.4 ± 19.6 (*n* = 706)	58.1 ± 19.3 (*n* = 288)	58.8 ± 19 (*n* = 470)				0.723
Positive symptoms	15.3 ± 6.5 (*n* = 711)	15.3 ± 6.8 (*n* = 291)	15.1 ± 6.6 (*n* = 474)				0.661
Negative symptoms	14.7 ± 6.3 (*n* = 708)	13.3 ± 6.6 (*n* = 290)	14.2 ± 6.3 (*n* = 470)				0.109
General psychopathology	30.3 ± 9.8 (*n* = 710)	29.3 ± 9.4 (*n* = 290)	29.5 ± 9.5 (*n* = 472)				0.867
BCSS							
Negative-self	10.4 ± 4.4 (*n* = 476)	10.3 ± 4.4 (*n* = 280)	10.3 ± 4.4 (*n* = 432)	6.7 ± 3.2 (*n* = 356)	<0.001	<0.001	0.918
Negative-others	10.6 ± 4.2 (*n* = 476)	10.4 ± 4.1 (*n* = 280)	10.6 ± 4.2 (*n* = 432)	8.5 ± 3.2 (*n* = 356)	<0.001	<0.001	0.460
K-PERS-I							
Total		5.4 ± 4.7 (*n* = 295)		0.0 ± 4.5 (*n* = 320)	<0.001		
Paternal age at birth		−0.2 ± 0.4 (*n* = 393)		−0.3 ± 0.4 (*n* = 354)	0.016		
Obstetric complications		0.2 ± 0.5 (*n* = 363)		0.0 ± 0.3 (*n* = 355)	<0.001		
Parental SES		0.4 ± 0.5 (*n* = 396)		0.5 ± 0.5 (*n* = 353)	0.686		
Urbanicity		−1.4 ± 1.5 (*n* = 398)		−1.1 ± 1.5 (*n* = 321)	0.023		
Childhood adversity		3.4 ± 1.9 (*n* = 390)		1.1 ± 1.9 (*n* = 355)	<0.001		
Recent life events		3.0 ± 3.5 (*n* = 337)		0.0 ± 3.3 (*n* = 356)	<0.001		
ETI-SR							
Total			5.7 ± 4.8 (*n* = 474)	3.0 ± 3.2 (*n* = 356)		<0.001	
General trauma			1.7 ± 1.8 (*n* = 475)	0.9 ± 1.2 (*n* = 356)		<0.001	
Physical punishment			1.7 ± 1.7 (*n* = 475)	1.4 ± 1.6 (*n* = 356)		0.006	
Emotional abuse			1.7 ± 1.7 (*n* = 475)	0.5 ± 1.1 (*n* = 356)		<0.001	
Sexual event			0.6 ± 1.2 (*n* = 476)	0.2 ± 0.6 (*n* = 356)		<0.001	

a SSDs with K-PERS-I (*n* = 280 ~ 398) versus HCs (*n* = 320 ~ 356); ^b^SSDs with ETI-SR (*n* = 432 ~ 476) versus HCs (*n* = 320 ~ 356); ^c^SSDs with K-PERS-I (*n* = 280 ~ 398) versus SZ with ETI-SR (*n* = 432 ~ 476); 269 SSDs patients with K-PERS-I group (*n* = 295) are overlapping with SSDs patients with ETI group (*n* = 474).

*Note*: BCSS, Brief Core Schema Scales; DI, duration of illness; DUP, duration of untreated psychosis; ETI-SR, Early Trauma Inventory Self Report; HCs, healthy controls; K-PERS-I, Korea Polyenvironmental Risk Score-I; NUH, National University Hospital; PANSS, Positive and Negative Syndrome Scale; PNOS, psychotic disorder not otherwise specified; SES, socioeconomic Status; SSDs, schizophrenia spectrum disorders; SSDs, schizophrenia spectrum disorders; All values were mean ± standard deviation (SD) unless otherwise specified.

### AUC and liability R^2^ for PRS-SZ versus PRS-SZ and K-PERS-I/ETI-SR

The PRS-SZ alone showed an AUC of 0.724 and a liability *R*^2^ of 8.7% (Supplementary Figure 3). However, when incorporating both PRS-SZ and K-PERS-I, we observed an AUC of 0.832 and a liability *R*^2^ of 22.6%. Combining PRS-SZ and ETI-SR resulted in moderate values, with an AUC of 0.766 and a liability *R*^2^ of 12.8% (Table 2). The SNP heritability for SZ calculated from the Korean-specific LD scores and EAS summary statistics was 0.20 ± 0.01.

**Table 2.** AUC and liability *R*^2^ of PRS-SZ versus PRS-SZ and K-PERS-I/ETI-SR on case–control status

Models	Total (SSDs/HCs)	AUC	Liability *R*^2^
PRS-SZ	1073 (717/356)	0.724	0.087
PRS-SZ + K-PERS-I + PRS-SZ * K-PERS-I	615 (295/320)	0.832	0.226
PRS-SZ + ETI-SR + PRS-SZ * ETI-SR	830 (474/356)	0.766	0.128

All variables were adjusted for age, sex, and education.

*Note:* AUC, area under the receiver operating curve; ETI-SR, Early Trauma Inventory Self Report; HCs, healthy controls; K-PERS-I, Korea Polyenvironmental Risk Score-I; PRS-SZ, Polygenic Risk Score-Schizophrenia; SSDs, schizophrenia spectrum disorders.

### Gene–environment correlation

We tested for correlations between PRS-SZ and K-PERS-I subdomains. No significant correlations were found in the patient group (Supplementary Table 1).

### Main and interaction effects of PRS-SZ and K-PERS-I/ETI-SR on case–control status

**Figure 1.**
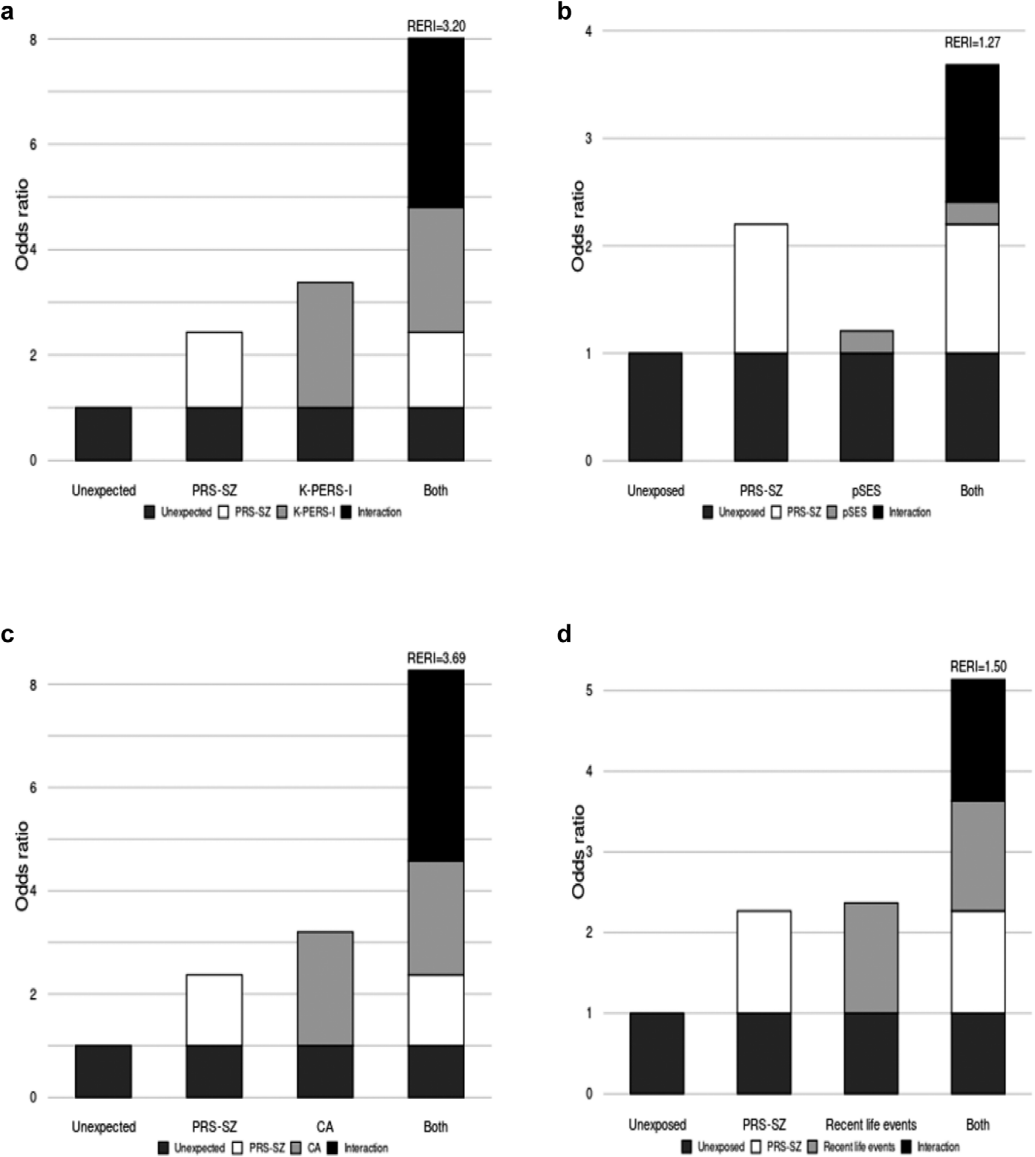
(a) Additive effects of PRS-SZ and K-PERS-I, (b) additive effects of PRS-SZ and pSES, (c) additive effects of PRS-SZ and CA, and (d) additive effects of PRS-SZ and Recent life events. *Note*: CA, childhood adversity; K-PERS-I, Korea Polyenvironmental Risk Score-I; PRS-SZ, Polygenic Risk Score-Schizophrenia; pSES, parental socioeconomic status; RERI, relative excess risk due to interaction.

When using the K-PERS-I as an environmental measure, the main effects in all four models were significantly associated with case–control status (PRS-SZ: adjusted OR = 2.20, 95% CI = 1.74–2.80, *p* = 1.68 × 10^−11^, K-PERS-I: adjusted OR = 3.26, 95% CI = 2.66–4.05, *p* = 7.41 × 10^−28^, PRS-SZ + K-PERS-I: adjusted OR = 3.39, 95% CI = 2.73–4.25, *p* = 7.01 × 10^−27^, PRS-SZ + K-PERS-I + PRS-SZ × K-PERS-I: adjusted OR = 3.37, 95% CI = 2.71–4.25, *p* = 3.74 × 10^−26^). No significant multiplicative interaction was observed, whereas a significant additive interaction was identified with a RERI of 3.20 (95% CI: 0.29–6.12, *p* = 0.015) (Figure 1a). Nagelkerke’s *R*^2^ values were highest for the independent and interaction models. Simulation using the standardized regression coefficients for the PRS-SZ by K-PERS-I multiplicative interaction yielded a power estimate of 5.6%. It was estimated that a minimum sample size of 180,000 individuals would be required to achieve 80% power. In contrast, for the additive interaction term, the power was approximately 88%, and the minimum sample size required to achieve 80% power was 520 individuals. When using ETI-SR as the environmental measure, all four models showed significant results, but no significant interaction effects were identified. Notably, the ORs for ETI-SR associated with SZ risk were lower than those observed for K-PERS-I (Table 3).

**Table 3.** Main and interaction effects of PRS-SZ and K-PERS-I/ETI-SR on case–control status

Models	Total (SSDs/HCs)	PRS-SZ		Environmental measures		Multiplicative interaction		Additive interaction		Nagelkerke’s *R*^2^
		Adjusted OR (95% CI)	p-value	Adjusted OR (95% CI)	p-value	Adjusted OR (95% CI)	p-value	RERI (95% CI)	p-value	
PRS-SZ	615 (295/320)	2.20 (1.74–2.80)	1.68 × 10^−11^	–	–	–	–	–	–	0.161
K-PERS-I		–	–	3.26 (2.66–4.05)	7.41 × 10^−28^	–	–	–	–	0.350
PRS-SZ + K-PERS-I		2.43 (1.85–3.24)	1.28 × 10^−10^	3.39 (2.73–4.25)	7.01 × 10^−27^	–	–	–	–	0.417
PRS-SZ + K-PERS-I + PRS-SZ × K-PERS-I		2.43 (1.85–3.24)	1.33 × 10^−10^	3.37 (2.71–4.25)	3.74 × 10^−26^	0.98 (0.76–1.25)	0.849	3.20 (0.29–6.12)	0.015	0.417
PRS-SZ	830 (474/356)	2.31 (1.89–2.82)	3.76 × 10^−16^	–	–	–	–	–	–	0.187
ETI-SR		–	–	1.93 (1.62–2.30)	1.47 × 10^−13^	–	–	–	–	0.173
PRS-SZ + ETI-SR		2.40 (1.94–2.97)	4.38 × 10^−16^	1.99 (1.65–2.38)	1.74 × 10^−13^	–	–	–	–	0.273
PRS-SZ + ETI-SR + PRS-SZ × ETI-SR		2.40 (1.94–2.96)	6.06 × 10^−16^	1.97 (1.64–2.38)	1.11 × 10^−12^	0.97 (0.80–1.17)	0.731	1.20 (−0.03–2.43)	0.900	0.273

All variables were adjusted for age, sex, and education.

*Note:* ETI-SR, Early Trauma Inventory Self Report; HCs, healthy controls; K-PERS-I, Korea Polyenvironmental Risk Score-I; OR, odd ratio; PRS-SZ, polygenic risk score-schizophrenia; RERI, relative excess risk due to interaction; SSDs, schizophrenia spectrum disorders.

**Figure 2.**
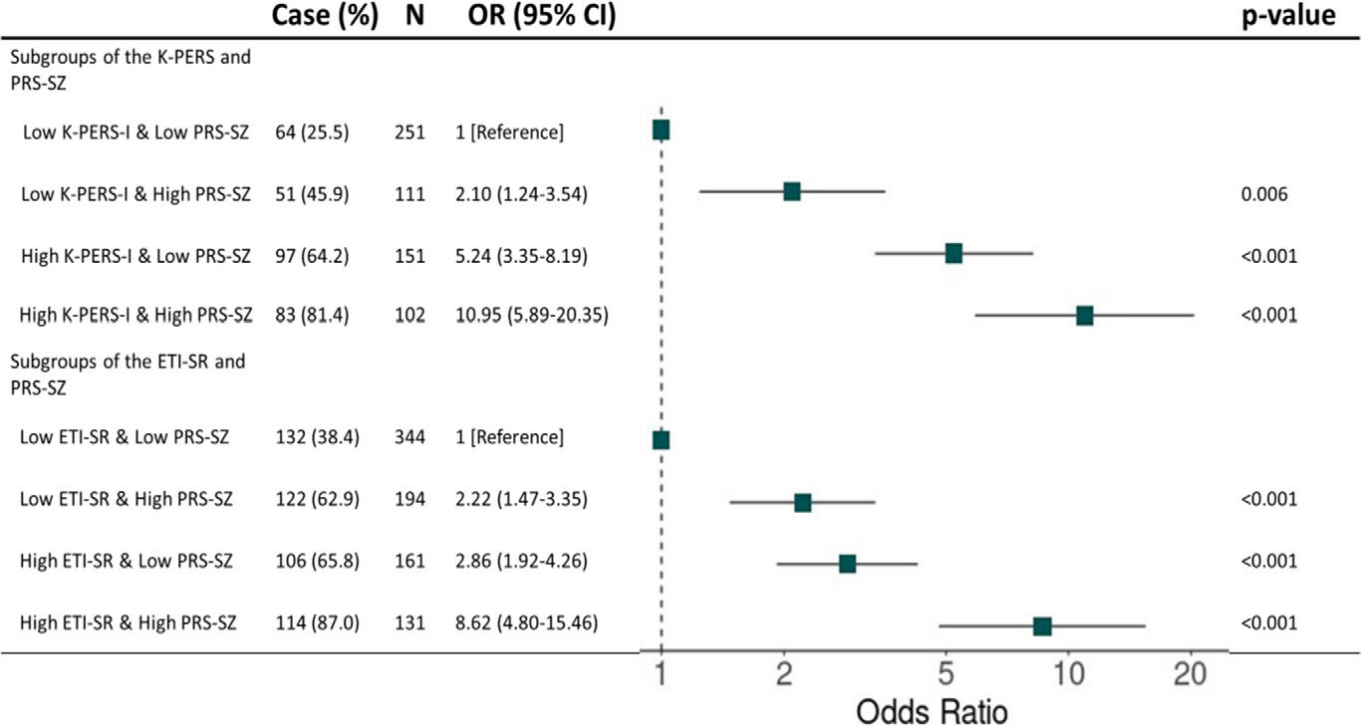
Regression results of subgroups of the K-PERS-I /ETI-SR and PRS-SZ were divided with a 75% cutoff of the control group. *Note*: ETI-SR, Early Trauma Inventory Self Report; K-PERS-I, Korea Polyenvironmental Risk Score-I; N, number; OR, odd ratio; PRS-SZ, Polygenic Risk Score-Schizophrenia.

In additional analyses using dichotomized PRS-SZ and K-PERS-I/ETI-SR with thresholds set at 75% of the HC group, the subgroup with high K-PERS-I and high PRS-SZ showed the highest OR (10.95, 95% CI = 5.89–20.35, *p* < 0.001) compared with the other three subgroups. For the subgroup with high ETI-SR and high PRS-SZ, the pattern was generally similar, but the OR was lower compared with the subgroup with a high K-PERS-I and high PRS-SZ (Figure 2). Sensitivity analyses using thresholds of 50% and 25% of the HC group showed similar results (Supplementary Figure 4). We found no different results between genders (Supplementary Table 2). Further analyses including three covariates (age, sex, and education) *GEss showed no significant results in all four models (Supplementary Table 3). AIC and BIC values of the new models assessing model fit were increased compared to the original models (Supplementary Table 4).

When using the K-PERS-I subdomains in the interaction models, both PRS-SZ and each subdomain were significantly associated with SZ risk. However, a significant multiplicative interaction with PRS-SZ was observed for pSES only (adjusted OR = 1.39, 95% CI =1.17–1.66, *p* = 2.70 × 10^−4^, FDR = 0.002). Notably, its Nagelkerke’s *R*^2^ value was 19.2%. Regarding additive interactions, PRS-SZ showed significant associations with pSES (RERI = 1.27, 95% CI = 0.32–2.23, *p* = 0.005, FDR = 0.013), childhood adversity (RERI = 3.69, 95% CI = 1.08–6.30, *p* = 0.003, FDR = 0.013), and recent life events (RERI = 1.50, 95% CI = 0.13–2.88, *p* = 0.016, FDR = 0.027) (Figure 1a–d, Supplementary Table 5). The additive interaction between PRS-SZ and pSES showed a power of 97.3%, with a minimum sample size of 450 individuals required to achieve 80% power. When using the ETI-SR subdomains, similar main effects were observed; however, no significant interactions remained after FDR correction (Supplementary Table 6).

### Main and interaction effects of PRS-SZ and K-PERS-I/ETI-SR on the PANSS and BCSS in patients with SSDs

For the PANSS, an interaction model with the K-PERS-I showed neither main effects nor additive interaction. However, in the model with the ETI-SR, PRS-SZ was significantly associated with the total score (Beta = 2.66, SD = 1.26, *p* = 0.036, FDR = 0.036). In contrast, the total ETI-SR score was only associated with positive symptom score (Beta = 0.93, standard deviation (SD) = 0.28, *p* = 0.001, FDR = 0.003). No significant interaction was evident. For the BCSS, the interaction model with the K-PERS-I showed neither main effects nor interaction. However, in the model with the ETI-SR, the total ETI-SR score was significantly associated with negative-self (Beta = 0.99, SD = 0.19, *p* = 4.14 × 10^−7^, FDR = 4.14 × 10^−7^) and negative-others (Beta = 1.38, SD = 0.18, *p* = 1.34 × 10^−13^, FDR = 2.68 × 10^−13^) (Table 4).

**Table 4.** Main and interaction effects of PRS-SZ and K-PERS-I/ETI-SR on the PANSS or BCSS in patients with SSDs

Phenotypes	Models	N	PRS-SZ				Environmental measures				Additive interaction				*R*^2^
			Beta	SD	p-value	FDR value	Beta	SD	p-value	FDR value	Beta	SD	p-value	FDR value	
PANSS															
Total	PRS-SZ + K-PERS-I + PRS-SZ × K-PERS-I	288	1.88	1.86	0.313	0.313	0.67	1.31	0.610	0.610	−1.49	1.51	0.320	0.320	0.069
	PRS-SZ + ETI-SR + PRS-SZ × ETI-SR	470	2.66	1.26	0.036	0.036	1.50	0.80	0.061	0.061	0.17	0.92	0.850	0.850	0.061
PANSS subdomains															
Positive symptoms	PRS-SZ + K-PERS-I + PRS-SZ × K-PERS-I	291	0.13	0.65	0.848	0.848	0.64	0.46	0.164	0.482	−0.39	0.53	0.460	0.460	0.082
	PRS-SZ + ETI-SR + PRS-SZ × ETI-SR	474	0.46	0.44	0.295	0.295	0.93	0.28	0.001	0.003	−0.15	0.32	0.640	0.960	0.052
Negative symptoms	PRS-SZ + K-PERS-I + PRS-SZ × K-PERS-I	290	0.99	0.64	0.121	0.363	−0.45	0.45	0.321	0.482	−0.73	0.52	0.160	0.460	0.038
	PRS-SZ + ETI-SR + PRS-SZ × ETI-SR	470	0.98	0.43	0.023	0.060	−0.18	0.27	0.508	0.508	−0.01	0.31	0.990	0.990	0.023
General psychopathology	PRS-SZ + K-PERS-I + PRS-SZ × K-PERS-I	290	0.77	0.89	0.389	0.584	0.36	0.63	0.569	0.569	−0.62	0.73	0.400	0.460	0.080
	PRS-SZ + ETI-SR + PRS-SZ × ETI-SR	472	1.28	0.62	0.040	0.060	0.72	0.39	0.069	0.104	0.28	0.45	0.540	0.960	0.085
BCSS															
Negative-self	PRS-SZ + K-PERS-I + PRS-SZ × K-PERS-I	280	0.09	0.44	0.835	0.879	0.55	0.31	0.075	0.150	−0.12	0.36	0.745	0.759	0.044
	PRS-SZ + ETI-SR + PRS-SZ × ETI-SR	432	−0.01	0.31	0.988	0.988	0.99	0.19	4.14 × 10^−7^	4.14 × 10^−7^	0.49	0.23	0.032	0.064	0.103
Negative-others	PRS-SZ + K-PERS-I + PRS-SZ × K-PERS-I	280	−0.06	0.42	0.879	0.879	0.37	0.29	0.206	0.206	0.10	0.34	0.759	0.759	0.015
	PRS-SZ + ETI-SR + PRS-SZ × ETI-SR	432	0.10	0.29	0.729	0.988	1.38	0.18	1.34 × 10^−13^	2.68 × 10^−13^	0.27	0.21	0.216	0.216	0.146

All variables were adjusted for age, sex, and education.

*Note:* BCSS, Brief Core Schema Scales; ETI-SR, Early Trauma Inventory Self Report; FDR, false discovery rate; K-PERS-I, Korea Polyenvironmental Risk Score-I; N, number of patients with SSDs; PANSS, Positive and Negative Syndrome Scale; PRS-SZ, polygenic risk score-schizophrenia; SSDs, schizophrenia spectrum disorders.

Additional analyses of the PANSS using K-PERS-I subdomains revealed no significant main effect of PRS-SZ, and no interaction effects remained significant after FDR correction. For the BCSS, no main effect of the PRS-SZ was evident, whereas CA was significantly associated with both negative-self (Beta = 0.72, SD = 0.27, *p* = 0.008, FDR = 0.008) and negative-others (Beta = 1.04, SD = 0.25, *p* < 0.001, FDR < 0.001). Intriguingly, a significant positive interaction between PRS-SZ and obstetric complications was identified in association with negative-others (Beta = 0.55, SD = 0.19, *p* = 0.004, FDR = 0.008) (Supplementary Table 7). In the model with ETI-SR subdomains, PRS-SZ was significantly associated with total, negative symptom, and general psychopathology scores; several subdomains of the ETI-SR were significantly associated with total, positive symptom, and general psychopathology scores. No significant interaction was noted. For the analyses with the BCSS, significant associations with negative-self and negative-others were only noted for the ETI-SR subdomains (Supplementary Table 8). After imputation, the results for Tables 3 and 4 were the same (Supplementary Tables 9–11).

## Discussion

Studies of GEIs may partly explain why SZ only develops in some people who experience relevant environmental exposures. Here, we investigated associations of PRS-SZ and total K-PERS-I and their interactions with case–control status and clinical phenotypes. Additionally, we investigated these associations using the K-PERS-I subdomains, as well as the total and subdomains of the ETI-SR.

In the present study, the PRS-SZ explained approximately 9% of SZ risk, which is similar to the reported 7% of the variance on the liability scale (Ripke et al., 2014) but higher than other reported rates of ~3% (Lam et al., 2019) or 3.4% (Agerbo et al., 2015). When the K-PERS-I and interaction term (PRS-SZ × K-PERS-I) were combined, the proportion of explained risk increased to 22.6%. This finding suggests that approaches considering both genetic and environmental factors and their interactions could enhance the accuracy of SZ risk prediction. Regarding the ORs for PRS-SZ and K-PERS-I in Table 3, the values of the K-PERS-I from the environmental model and two combined models (PRS-SZ + K-PERS-I and PRS-SZ + K-PERS-I + PRS-SZ × K-PERS-I) were greater than the values of PRS-SZ from the genetic model. These findings indicate that the contributions of environmental factors and the combined effects of genetic and environmental factors in association with SSD risk are greater than the contributions of genetic effects alone. However, this pattern was not observed when the ETI-SR was regarded as an environmental factor. The ORs of the ETI-SR from the environmental model and two combined models were smaller than the ORs of PRS-SZ from the genetic model. Moreover, the ORs of the ETI-SR were smaller than the ORs of the K-PERS-I. These findings suggest that using the K-PERS-I, a more comprehensive tool that measures multiple environmental factors, may be more effective than using the ETI-SR for predicting SSD risk. However, this interpretation should be approached with caution, as the sample sizes between the two datasets differ.

For the interaction with the K-PERS-I, we observed contrasting results between multiplicative versus additive models. Considering that an additive model can provide a superior representation of biological synergy and inform public health decisions within the sufficient cause framework (Kendler & Gardner 2010; Rothman, 1976), the result of the additive model needs to be more highlighted. It suggests that the combined effect of genetic and environmental risk factors is greater than the sum of their individual effects, which is in align with the findings of GEI studies using the ES-SZ (Pries, Dal Ferro, et al., 2020) and ERS (Mas et al., 2020). The implication of this synergistic interaction should be incorporated into psychoeducation and public health policy. The lack of significant interactions with the ETI-SR in both models may be due to different characteristics of the two measures: several subdomains of the K-PERS-I capture pre/perinatal or chronic exposures that are likely to affect or interact with genetic risk, while ETI-SR focuses on acute events, which may act independently of genetic predisposition. This emphasizes the need to consider the duration and nature of environmental exposures in GEI studies. One meta-analysis reported that the current four studies on the interaction between PRS and childhood adversity yielded inconsistent findings (Woolway et al., 2022). Interestingly, when PRS-SZ and K-PERS-I/ETI-SR were dichotomized using a threshold of 75% of the control group value, the subgroups with high K-PERS-I/ETI-SR and high PRS-SZ showed the highest ORs, approximately 9–10, compared with the other three subgroups. If replicated in population-based cohort studies, these results could have important implications for the early identification of individuals at risk of developing SSDs and the provision of more intensive psychosocial interventions that target modifiable environmental factors in patients with SSDs.

When using the subdomains of the K-PERS-I, we found a significant interaction, departing from both multiplicativity and additivity, between PRS-SZ and pSES in association with SSD risk, indicating a synergistic effect. This suggests that the impact of pSES on SSD risk is moderated by PRS-SZ. To our knowledge, this is the first report demonstrating a significant interaction between pre-existing genetic liability and pSES in association with SSD risk, although a negative finding has been documented (Agerbo et al., 2015). Notably, Hatzimanolis et al., 2020 found a significant interaction between familial risk of psychosis and pSES in terms of influencing social premorbid adjustment in childhood among patients with psychosis. There could be several explanations for this synergistic effect; poor pSES may lead to lower education, neighborhood deprivation, and health inequity relative to an affected individual’s siblings, all of which are factors related to mental health (González et al., 2023; Luo, van Grieken, Yang-Huang, van den Toren, & Raat, 2022). Moreover, the cumulative effects of these interconnected factors may contribute to a synergistic effect, or the pSES may have exerted detrimental effects on SSD-associated mutants to produce more dysfunctional changes through epigenetic regulation. We identified significant additive interactions between PRS-SZ and both childhood adversity and recent life events. A synergistic interaction result between PRS-SZ and childhood adversity is in line with previous studies (Aas et al., 2023; Guloksuz et al., 2019). Furthermore, given the interaction between PRS-major depressive disorder and childhood trauma (Peyrot et al., 2014), it reinforces the idea that genetic risk and early-life stressors can act together to increase susceptibility to mental health disorders. Similarly, recent life events, like stress or loss, can trigger or exacerbate genetic susceptibility to SZ. However, a study found that ES-SZ moderated the link between stressful life events and mental health, while PRS-SZ did not (Pries et al., 2020), highlighting the complexity of GESs. For the contribution of the K-PERS-I subdomains to SZ risk, all six factors were significant. Given that high paternal age is a risk factor for SZ and the average marriage age is increasing these days, this should be considered in psychoeducation and public health policy. Unexpectedly, urbanicity, which is strongly associated with SZ risk in other populations (Plana-Ripoll, Pedersen, & McGrath, 2018), was found to be a protective factor. This may be explained by easy access to mental healthcare services (Park, Park, Kwon, Kang, & Noh, 2016) and lower rates of drug abuse (Jang et al., 2023) in Korea compared to Western countries.

In the model with the K-PERS-I, neither PRS-SZ nor K-PERS-I were associated with the PANSS or BCSS, but in the model with the ETI-SR, PRS-SZ and ETI-SR were associated with several items. These findings suggest that the K-PERS-I is not an appropriate tool for investigating its association with clinical phenotypes. Concerning the results with the model that included the ETI-SR, PRS-SZ was significantly associated with the total score of the PANSS. Studies using PRS-SZ have yielded conflicting findings regarding associations with the symptom dimensions of SZ. Evidence supporting an association between PRS-SZ and positive symptoms is scarce, whereas studies of negative symptoms have led to mixed results (Mistry, Harrison, Smith, Escott-Price, & Zammit, 2018; Ronald & Pain, 2018). Considering these inconclusive findings and the low prediction accuracy (~6%) of PRS-SZ for genetic variation in psychiatric phenotypes (Mistry et al., 2018), the utility of PRS-SZ in clinical settings appears limited. In contrast, ETI-SR was associated with positive symptoms, negative-self, and negative-others. Because negative evaluations about the self and others or negative self-concepts are associated with the formation of delusions and hallucinations (Garety, Kuipers, Fowler, Freeman, & Bebbington, 2001; Smith et al., 2006), this finding may provide insights about how CA is involved in developing positive symptoms. Additionally, albeit at an uncorrected level, a synergistic effect between PRS-SZ and ETI-SR in predicting negative-self was observed. Taken together with the case–control status results, the model with the K-PERS-I may be better in terms of discriminating case–control status, whereas the model with the ETI-SR may be more suitable for predicting symptomatology.

In the subdomain models, CA of the K-PERS-I was associated with negative-self and negative-others. The positive interaction of PRS-SZ and obstetric complications in predicting negative-others is intriguing in that the association with negative-others only manifested when both genetic and environmental factors played a role; it was absent when each set of factors was present in isolation. For the synergistic effect, it is possible that individuals with genetic liability and obstetric complications have a higher likelihood of exposure to negative life events, which may lead to the formation of negative-others. Regarding the results with the model that included subdomains of the ETI-SR, the patterns were similar to those in the model with total ETI-SR.

Although this was the first study of GEIs using the K-PERS-I, several limitations should be considered. First, careful interpretation of the results is required, given that the sample size may have been insufficient for detecting multiplicative interaction potentially leading to type II errors. However, it is of note that in terms of additive interaction, the sample size was adequate. In addition, although we addressed missing data in the total K-PERS-I or ETI-SR by imputation, caution is needed when interpreting comparisons between total scores and subdomains due to differing sample sizes. Second, the study’s cross-sectional design did not allow for an investigation of the dynamic nature of the GEIs over time, hindering causal inference. Future research should focus more extensively on prospective cohort studies, including samples at different time points. Third, as we excluded foreigners to participate, the ethnic homogeneity of the sample, comprising original residents of South Korea, limits the generalizability of the findings to other populations. Fourth, although widely used, the RERI has limitations. Since it is based on ratios, it cannot be directly interpreted as the combined effects of exposures (Zhao & Thompson, 2023). Fifth, K-PERS-I does not account for the interdependency of exposures like ES-SZ. Additionally, it excludes cannabis use and immigration, which may limit its ability to fully capture environmental influences on SZ risk and its applicability across diverse populations. Finally, the retrospective assessment of the K-PERS-I and ETI-SR may be associated with recall bias. Despite these limitations, this study has notable strengths, including being the first GEI study conducted in Korea and Asian countries and uniquely investigating and comparing the utilities of two environmental measures in the models.

In conclusion, using the aggregate scores of PRS-SZ and K-PERS-I, we did not observe any interactions in association with case–control status. However, when using the subdomains of the K-PERS-I or total and subdomains of the ETI-SR, several interactions were identified in association with case–control status and clinical phenotypes. These findings suggest that the aggregate scores of genetic and environmental measures, PRS-SZ and K-PERS-I, are unsuitable for investigating GEIs, which were instead detected with specific environmental measures. However, the contribution of the total K-PERS-I to case–control status was greater than the contribution of the total ETI-SR. There is a need for additional research concerning GEIs and modifiable environmental factors.

## Supporting information

Rami et al. supplementary material Rami et al. supplementary material

## Supplementary material

The supplementary material for this article can be found at http://doi.org/10.1017/S0033291725000753.
