# Supplementary material for: Associations of polygenic risk score, environmental factors, and their interactions with the risk of schizophrenia spectrum disorders
Source: Psychol Med. 2025 Apr 11;55:e111. doi: 10.1017/S0033291725000753 (PMC12094650; doi:10.1017/S0033291725000753)
Supplement: Rami et al. supplementary material [file S0033291725000753sup001.zip › Association of PRS and PERS with SZ-Suppl Figures-clean.docx]

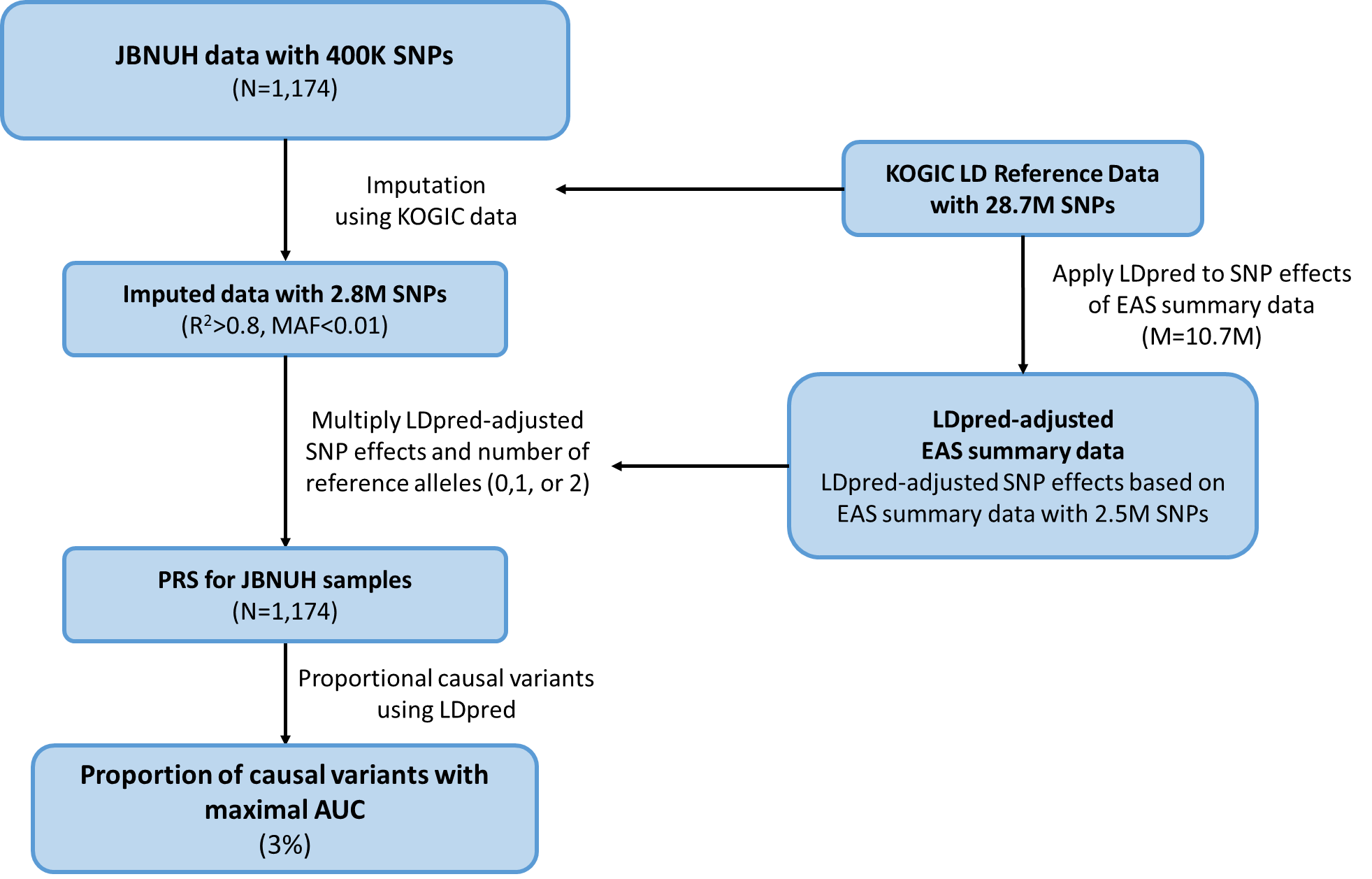


Supplementary Figure 1. Flowchart of PRS calculation

**Notes:** AUC: Area Under the receiver operating Curve, EAS: East Asian, JBNUH: Jeonbuk National University Hospital, KOGIC: Korean Genomics Center, LD: Linkage Disequilibrium, LDpred: Linkage Disequilibrium prediction, MAF: Minor Allele Frequency, PRS: Polygenic Risk Score, SNPs: Single Nucleotide Polymorphisms.


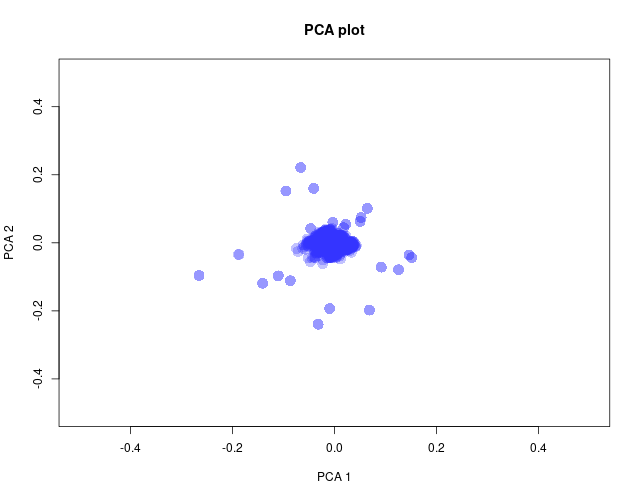

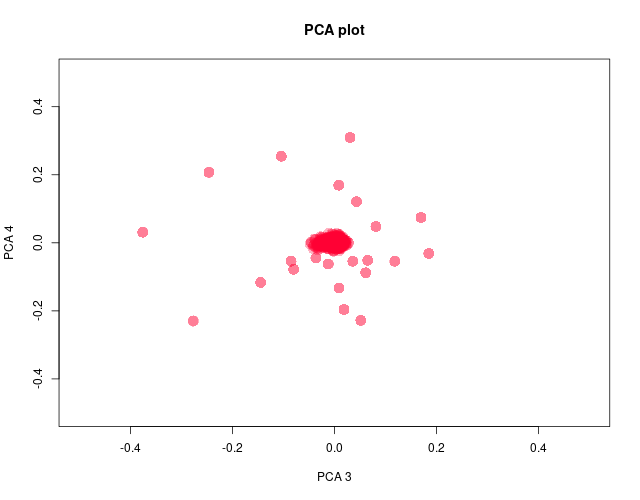


Supplementary Figure 2. Principal components analysis plot based on schizophrenia SNPs.

**Notes**: PCA: Principal Component Analysis, SNPs: Single Nucleotide Polymorphisms.


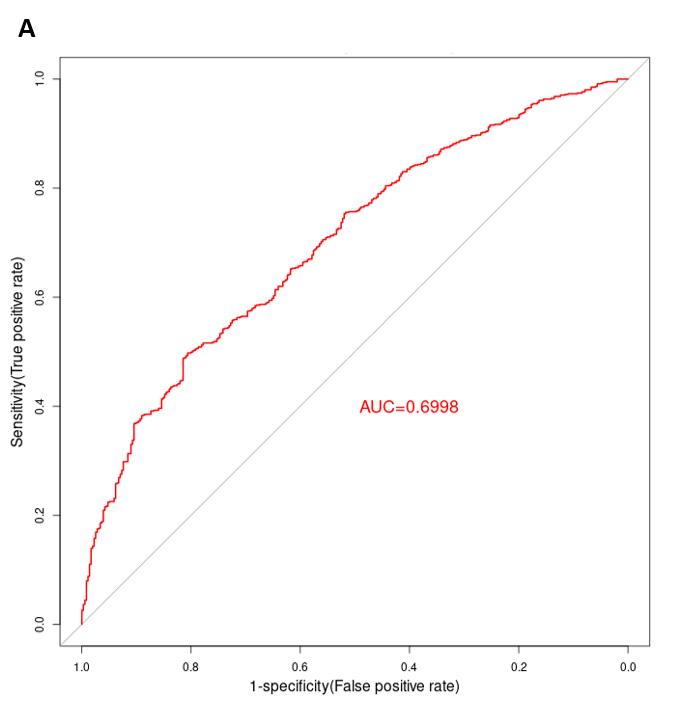

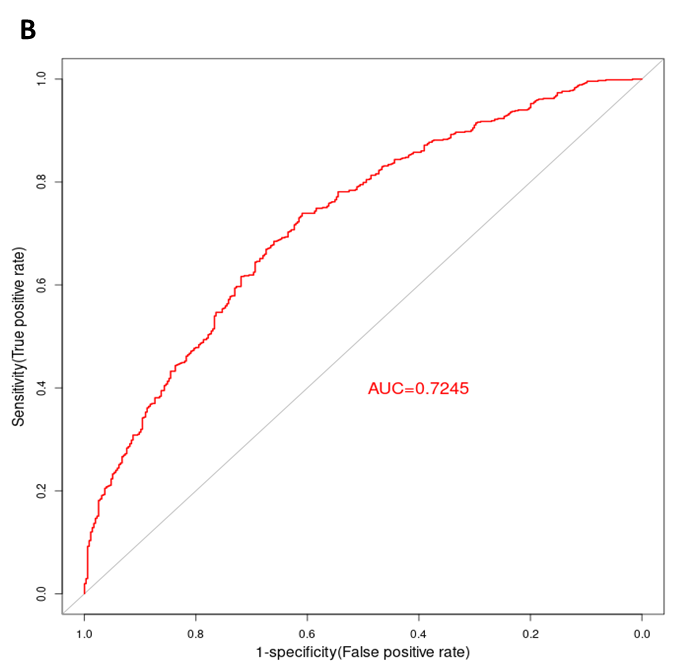


Supplementary Figure 3. The ROC curves representing the prediction accuracy of PRS for schizophrenia, A: unadjusted PRS-SZ, B: adjusted PRS-SZ for age, sex, and education.

**Notes**: AUC: Area Under the receiver operating Curve, PRS-SZ: Polygenic Risk Score- Schizophrenia, ROC: Receiver Operating Curve.


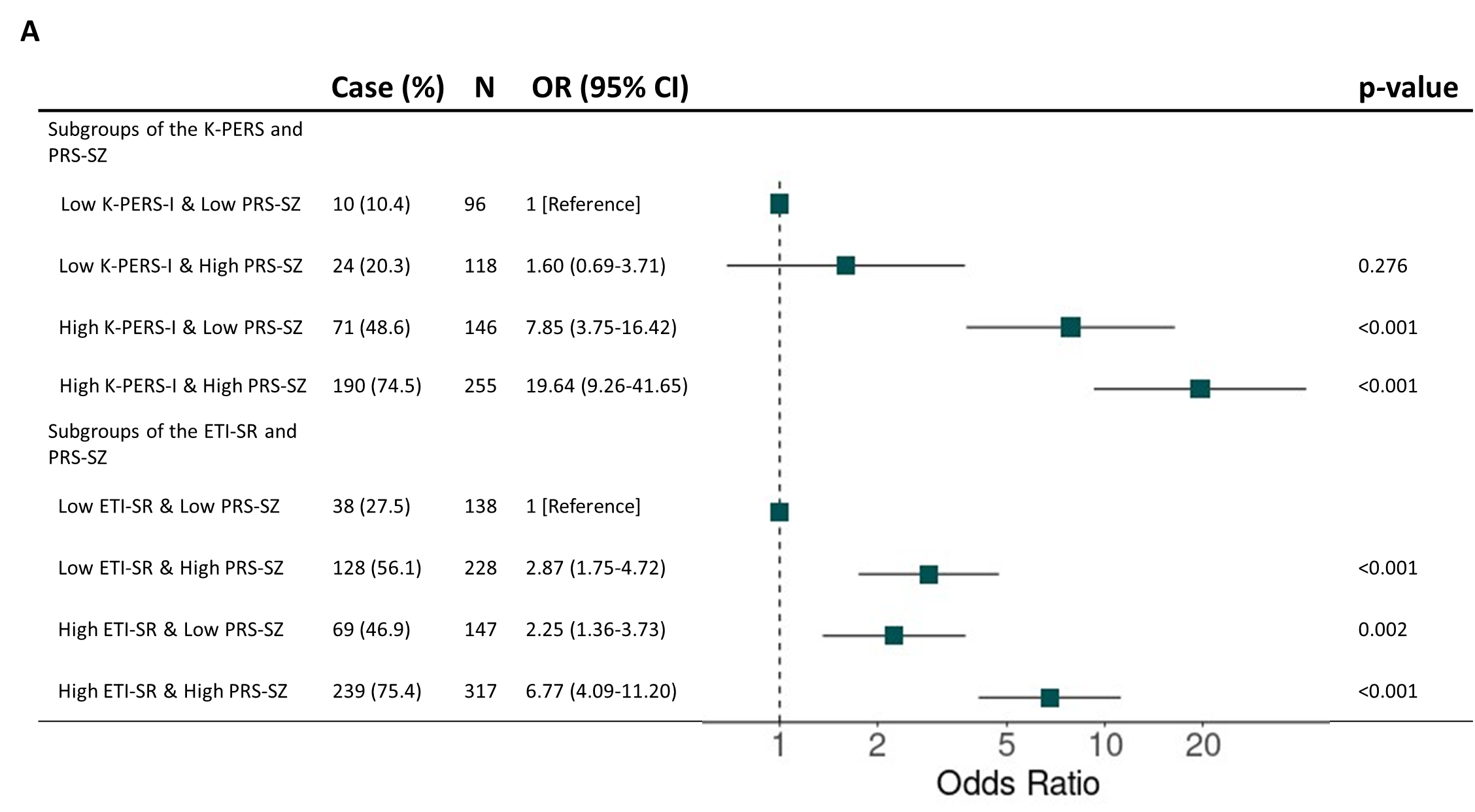


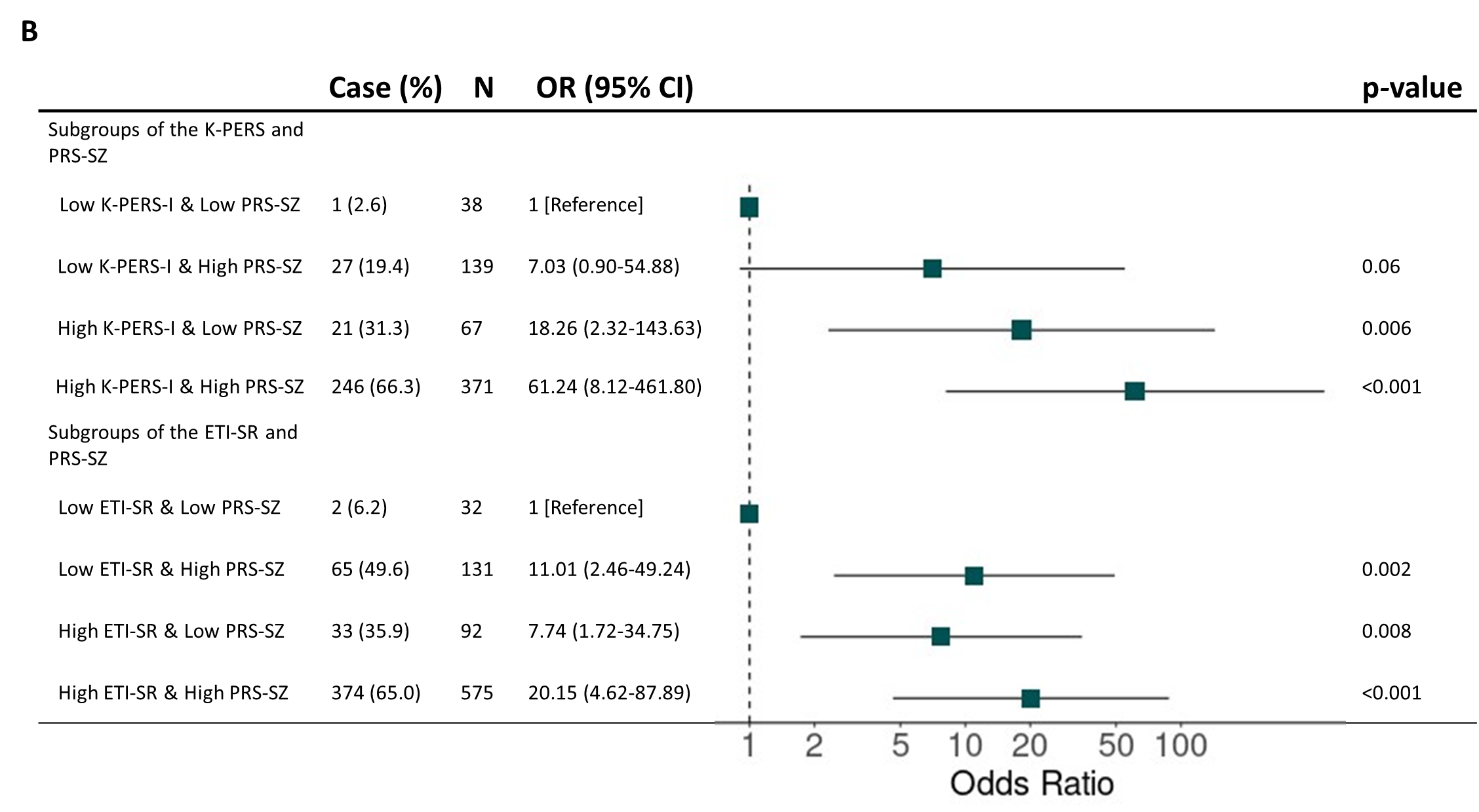


Supplementary Figure 4. Regression results with subgroups of the K-PERS-I / ETI-SR and PRS-SZ divided with 50% (A) and 25% (B) cut-off of the control group.

**Notes:** ETI-SR: Early Trauma Inventory Self Report, K-PERS-I: Korea Polyenvironmental Risk Score-I, OR: Odd Ratio, PRS-SZ: Polygenic Risk Score-Schizophrenia.
