# Supplementary material for: Associations of polygenic risk score, environmental factors, and their interactions with the risk of schizophrenia spectrum disorders
Source: Psychol Med. 2025 Apr 11;55:e111. doi: 10.1017/S0033291725000753 (PMC12094650; doi:10.1017/S0033291725000753)
Supplement: Rami et al. supplementary material [file S0033291725000753sup001.zip › Association of PRS and PERS with SZ-Suppl Tables-clean.docx]

Supplementary materials

**1. Definition of subdomains of the K-PERS-I and its scoring**

Paternal age at birth equal to or greater than 36 years old was scored as 0.5, and under 36 years old, as -0.5. Obstetric complications were identified based on whether the subject was born with a low birth weight (less than 2.5 kg) (2 for yes and 0 for no). Parental socioeconomic status during childhood (up to 12 years old) was classified as difficult (1) or good (0) based on parents’ education and occupation. Urbanicity was considered present (0) when a person was raised in a city for more than 50% of childhood (up to 12 years old). Rural living was given a score of 3. Childhood adversity encompasses emotional abuse, emotional neglect, physical abuse, and sexual abuse. If at least one trauma was present, it was scored as 4.5; otherwise, as 0. Adult life events evaluated whether ones experienced at least two adverse events (5.5 for yes and -2 for no) among living alone, financial hardship, and difficulties in social relationships and occupational or academic functioning at least 6 months prior to the development of psychotic symptoms (Jeon et al., 2022).

**2. Quality control of genetic data**

The genotype data were obtained by merging data from 528,213 Single Nucleotide Polymorphisms (SNPs) in 148 patients generated using the KORV 1.0 (Affymetrix, Santa Clara, CA, USA) and 580,748 SNPs in 1,026 individuals (670 patients and 356 healthy individuals) generated using the KORV 1.1 (Affymetrix, Santa Clara, CA, USA). The combined dataset consisted of 461,010 SNPs and 1,174 individuals. Following quality control analysis with the PLINK (v1.90) (Purcell et al., 2007), 437,367 SNPs in 1,174 individuals remained. The SNPs with minor allele frequencies <0.01, genotype calling rates <97%, Missing Rate Per Person > 5% and Hardy-Weinberg equilibrium p-values < 10^-6^, as well as strand-ambiguous SNPs were removed. After mapping to the UCSC hg19 genomic coordinates, principal component analysis using PLINK was performed to identify outliers (Supplementary Figure 2). Because of the need to match age, sex, and education between patients and controls, only the data of 717 patients was utilized for the calculation of PRS.

Supplementary Table 1. Gene-environment correlations between PRS-SZ and K-PERS-I subdomains in patients with SSDs

| Environmental exposure | Total  (Environmental exposure high/low) | PRS-SZ | |
| --- | --- | --- | --- |
|  |  | Adjusted OR (95% CI) | p-value |
| Paternal age at birth | 393 (106/287) | 0.83 (0.61-1.14) | 0.251 |
| Obstetric complications | 363 (28/335) | 0.70 (0.40-1.21) | 0.205 |
| Parental SES | 396 (177/219) | 0.87 (0.65-1.17) | 0.354 |
| Urbanicity | 398 (217/181) | 0.95 (0.70-1.30) | 0.752 |
| Childhood adversity | 390 (297/93) | 0.75 (0.53-1.06) | 0.099 |
| Recent life events | 337 (224/113) | 0.84 (0.60-1.17) | 0.300 |

All variables were adjusted for age, sex and education.

**Notes**: K-PERS-I: Korea Polyenvironmental Risk Score-I, OR: Odd Ratio, PRS-SZ: Polygenic Risk Score-Schizophrenia, SES: Socio-Economic Status, SSDs: Schizophrenia Spectrum Disorders.

Supplementary Table 2. Main and interaction effects of PRS-SZ and K-PERS-I/ETI-SR on case-control status in males and females

| **Males** | | | | | | | | | | |
| --- | --- | --- | --- | --- | --- | --- | --- | --- | --- | --- |
| Models | Total (SSDs/HCs) | PRS-SZ | | Environmental measures | | Multiplicative Interaction | | Additive interaction | | Nagelkerke’s R^2^ |
|  |  | Adjusted OR (95% CI) | p-value | Adjusted OR (95% CI) | p-value | Adjusted OR (95% CI) | p-value | RERI  (95% CI) | p-value |  |
| PRS-SZ | 303  (137/166) | 2.22 (1.59-3.11) | 3.58 x 10^-06^ | - | - | - | - |  |  | 0.145 |
| K-PERS-I |  | - | - | 3.1 (2.32-4.15) | 2.85 x 10^-14^ | - | - |  |  | 0.325 |
| PRS-SZ + K-PERS-I |  | 2.49 (1.67-3.70) | 6.77 x 10^-06^ | 3.23 (2.38-4.38) | 6.48 x 10^-14^ | - | - |  |  | 0.399 |
| PRS-SZ + K-PERS-I + PRS-SZ * K-PERS-I |  | 2.52 (1.68-3.76) | 6.70 x 10^-06^ | 3.2 (2.36-4.35) | 1.06 x 10^-13^ | 0.9 (0.62-1.29) | 0.568 | 3.20 (0.29-6.12) | 0.016 | 0.400 |
| PRS-SZ | 408  (230/178) | 2.24 (1.69-2.96) | 1.91 x 10^-08^ | - | - | - | - |  |  | 0.160 |
| ETI-SR |  | - | - | 1.74 (1.37-2.21) | 5.64 x 10^-06^ | - | - |  |  | 0.124 |
| PRS-SZ + ETI-SR |  | 2.25 (1.68-3) | 4.35 x 10^-08^ | 1.74 (1.35-2.23) | 1.32 x 10^-05^ | - | - |  |  | 0.219 |
| PRS-SZ + ETI-SR + PRS-SZ * ETI-SR |  | 2.25 (1.68-3) | 4.09 x 10^-08^ | 1.75 (1.36-2.25) | 1.56 x 10^-05^ | 1.04 (0.79-1.37) | 0.757 | 1.19 (-0.03-2.43) | 0.900 | 0.219 |
| **Females** | | | | | | | | | | |
| PRS-SZ | 312  (158/154) | 2.20 (1.57-3.08) | 4.97 x 10^-06^ | - | - | - | - |  |  | 0.176 |
| K-PERS-I |  | - | - | 3.53 (2.59-4.82) | 2.11 x 10^-15^ | - | - |  |  | 0.374 |
| PRS-SZ + K-PERS-I |  | 2.34 (1.57-3.49) | 2.84 x 10^-05^ | 3.61 (2.61-4.99) | 8.71 x 10^-15^ | - | - |  |  | 0.432 |
| PRS-SZ + K-PERS-I + PRS-SZ * K-PERS-I |  | 2.35 (1.58-3.51) | 2.58 x 10^-05^ | 3.65 (2.61-5.1) | 4.04 x 10^-14^ | 1.05 (0.74-1.47) | 0.794 | 3.20 (0.29-6.12) | 0.016 | 0.432 |
| PRS-SZ | 422  (244/178) | 2.47 (1.84-3.32) | 1.75 x 10^-09^ | - | - | - | - |  |  | 0.221 |
| ETI-SR |  | - | - | 2.15 (1.65-2.78) | 8.61 x 10^-09^ | - | - |  |  | 0.223 |
| PRS-SZ + ETI-SR |  | 2.69 (1.96-3.68) | 6.88 x 10^-10^ | 2.28 (1.73-2.99) | 3.92 x 10^-09^ | - | - |  |  | 0.333 |
| PRS-SZ + ETI-SR + PRS-SZ * ETI-SR |  | 2.67 (1.94-3.66) | 1.31 x 10^-09^ | 2.23 (1.68-2.96) | 2.61 x 10^-08^ | 0.93 (0.7-1.24) | 0.634 | 1.19 (-0.03-2.43) | 0.900 | 0.334 |

All variables were adjusted for age and education.

**Notes** ETI-SR: Early Trauma Inventory Self Report, HCs: Healthy Controls, K-PERS-I: Korea Polyenvironmental Risk Score-I, OR: Odd Ratio, PRS-SZ: Polygenic Risk Score-Schizophrenia, RERI: Relative Excess Risk due to Interaction, SSDs: Schizophrenia Spectrum Disorders.

Supplementary Table 3. Main and interaction effects of PRS-SZ and subdomains of the K-PERS-I on case-control status

| Models | Total (SSDs/HCs) | PRS-SZ | | Environmental measures | | Multiplicative interaction | | Additive interaction | | Nagelkerke’s R^2^ |
| --- | --- | --- | --- | --- | --- | --- | --- | --- | --- | --- |
|  |  | Adjusted OR (95% CI) | p-value | Adjusted OR (95% CI) | p-value | Adjusted OR (95% CI) | p-value | RERI  (95% CI) | p-value |  |
| PRS-SZ | 615 (295/320) | 0.55 (0.14-2.13) | 0.388 | - | - | - | - | - | - | 0.170 |
| K-PERS-I |  | - | - | 1.95 (0.4-9.4) | 0.406 | - | - | - | - | 0.358 |
| PRS-SZ + K-PERS-I |  | 1.26 (0.27-5.85) | 0.768 | 2.66 (0.5-14.14) | 0.252 | - | - | - | - | 0.425 |
| PRS-SZ + K-PERS-I + PRS-SZ * K-PERS-I |  | 1.42 (0.3-6.72) | 0.660 | 3.59 (0.64-20.14) | 0.147 | 0.76 (0.55-1.05) | 0.096 | -0.15 (-4.06-3.76) | 0.529 | 0.429 |
| PRS-SZ | 830 (474/356) | 1.13 (0.36-3.61) | 0.833 | - | - | - | - | - | - | 0.191 |
| ETI-SR |  | - | - | 1.83 (0.52-6.46) | 0.349 | - | - | - | - | 0.179 |
| PRS-SZ + ETI-SR |  | 1.22 (0.36-4.12) | 0.744 | 1.97 (0.53-7.31) | 0.310 | - | - | - | - | 0.283 |
| PRS-SZ + ETI-SR + PRS-SZ * ETI-SR |  | 1.34 (0.39-4.59) | 0.639 | 2.27 (0.6-8.56) | 0.227 | 0.86 (0.67-1.1) | 0.236 | 0.01 (-1.65-1.68) | 0.990 | 0.284 |

All variables were adjusted for age, sex, education and their interaction terms.

**Notes**: ETI-SR: Early Trauma Inventory Self Report, HCs: Healthy Controls, K-PERS-I: Korea Polyenvironmental Risk Score-I, OR: Odd Ratio, PRS-SZ: Polygenic Risk Score-Schizophrenia, SSDs: Schizophrenia Spectrum Disorders.

Supplementary Table 4. Evaluation of Main and Interaction Effects of PRS-SZ and K-PERS-I Subdomains on Case-Control Status Based on AIC

| Models | AIC / BIC  (with covariates) | AIC/ BIC  (with covariates’ interaction terms) | AIC / BIC difference |
| --- | --- | --- | --- |
| PRS-SZ | 782.37 / 804.48 | 783.64 / 819.01 | +1.27 / +14.53 |
| K-PERS-I | 674.28 / 696.39 | 675.30 / 710.68 | +1.02 / +14.29 |
| PRS-SZ + K-PERS-I | 632.91 / 659.44 | 639.59 / 692.65 | +6.69 / +33.22 |
| PRS-SZ + K-PERS-I + PRS-SZ * K-PERS-I | 634.87 / 665.82 | 638.75 / 696.23 | +3.88 / +30.41 |
| PRS-SZ | 1019.45 / 1043.06 | 1022.14 / 1059.91 | +2.69 / +16.85 |
| ETI | 1029.47 / 1053.08 | 1030.98 / 1068.75 | +1.51 / +15.68 |
| PRS-SZ + ETI | 957.35 / 985.68 | 961.60 / 1018.26 | +4.25 / +32.58 |
| PRS-SZ + ETI + PRS-SZ * ETI | 959.24 / 992.29 | 962.20 / 1023.58 | +2.97 / +31.29 |

All variables were adjusted for age, sex, education and their interaction terms.

**Notes**:AIC: Akaike Information Criterion, BIC: Bayesian Information Criterion, ETI-SR: Early Trauma Inventory Self Report, HCs: Healthy Controls, K-PERS-I: Korea Polyenvironmental Risk Score-I, OR: Odd Ratio, PRS-SZ: Polygenic Risk Score-Schizophrenia, SSDs: Schizophrenia Spectrum Disorders.

Supplementary Table 5. Main and interaction effects of PRS-SZ and subdomains of the K-PERS-I on case-control status

|  | Total (SSDs/HCs) | PRS-SZ | | | Environmental measure | | | Multiplicative interaction | | | Additive interaction | | | Nagelkerke’s R^2^ |
| --- | --- | --- | --- | --- | --- | --- | --- | --- | --- | --- | --- | --- | --- | --- |
|  |  | Adjusted OR (95% CI) | p-value | FDR value | Adjusted OR (95% CI) | p-value | FDR value | Adjusted OR (95% CI) | p-value | FDR value | RERI  (95% CI) | p-value | FDR value |  |
| PRS-SZ * Paternal age at birth | 747 (393/354) | 2.20 (1.79-2.74) | 3.06 x 10^-13^ | 9.18 x 10^-13^ | 1.25 (1.06-1.49) | 0.010 | 0.012 | 0.90 (0.77-1.05) | 0.180 | 0.540 | 0.03 (-0.47-0.52) | 0.460 | 0.460 | 0.164 |
| PRS-SZ * Obstetric complications | 718 (363/355) | 2.31 (1.85-2.90) | 2.28 x 10^-13^ | 9.18 x 10^-13^ | 1.32 (1.10-1.65) | 0.006 | 0.009 | 0.95 (0.77-1.18) | 0.614 | 0.649 | 0.26 (-0.52-1.04) | 0.254 | 0.318 | 0.185 |
| PRS-SZ * Parental SES | 749 (396/353) | 2.20 (1.78-2.74) | 5.12 x 10^-13^ | 1.02 x 10^-12^ | 1.21 (1.01-1.45) | 0.038 | 0.038 | 1.39 (1.17-1.66) | 2.70 x 10^-4^ | 0.002 | 1.27 (0.32-2.23) | 0.005 | 0.013 | 0.192 |
| PRS-SZ * Urbanicity | 719 (398/321) | 2.23 (1.80-2.78) | 7.71 x 10^-13^ | 1.16 x 10^-12^ | 0.68 (0.56-0.82) | 7.11 x 10^-5^ | 1.42 x 10^-4^ | 0.91 (0.76-1.09) | 0.312 | 0.582 | NA | NA | NA | 0.191 |
| PRS-SZ * Childhood adversity | 745 (390/355) | 2.37 (1.86-3.06) | 1.12 x 10^-11^ | 1.34 x 10^-11^ | 3.20 (2.67-3.87) | 1.70 x 10^-34^ | 1.02 x 10^-33^ | 1.09 (0.90-1.32) | 0.388 | 0.582 | 3.69 (1.08-6.30) | 0.003 | 0.013 | 0.408 |
| PRS-SZ * Recent life events | 693 (337/356) | 2.27 (1.79-2.90) | 4.04 x 10^-11^ | 4.04 x 10^-11^ | 2.37 (1.98-2.84) | 3.86 x 10^-21^ | 1.16 x 10^-20^ | 0.96 (0.79-1.15) | 0.649 | 0.649 | 1.50 (0.13-2.88) | 0.016 | 0.027 | 0.334 |

All variables were adjusted for age, sex and education.

**Notes**: FDR: False discovery rate, HCs: Healthy Controls, K-PERS-I: Korea Polyenvironmental Risk Score-I, NA: Not Applicable (because of OR ＜1), OR: odd ratio, PRS-SZ: Polygenic Risk Score-Schizophrenia, RERI: Relative Excess Risk due to Interaction, SES: Socio-Economic Status, SSDs: Schizophrenia Spectrum Disorders.

Supplementary Table 6. Main and interaction effects of PRS-SZ and subdomains of the ETI-SR on case-control status

|  | Total (SSDs/HCs) | PRS-SZ | | | Environmental measure | | | | Multiplicative Interaction | | | | Additive interaction | | | | Nagelkerke’s R^2^ |
| --- | --- | --- | --- | --- | --- | --- | --- | --- | --- | --- | --- | --- | --- | --- | --- | --- | --- |
|  |  | Adjusted OR (95% CI) | p-value | FDR value | Adjusted OR (95% CI) | p-value | | FDR value | | Adjusted OR (95% CI) | p-value | FDR value | | RERI  (95% CI) | p-value | FDR value |  |
| PRS-SZ * General trauma | 831 (475/356) | 2.41 (1.96-2.98) | 2.25 x 10^-16^ | 8.02 x 10^-16^ | 1.76 (1.47-2.14) | | 3.05 x 10^-9^ | 6.10 x 10^-9^ | | 0.97 (0.80-1.18) | 0.750 | 0.855 | | 0.94 (-0.17-2.04) | 0.048 | 0.096 | 0.249 |
| PRS-SZ * Physical punishment | 831 (475/356) | 2.32 (1.90-2.85) | 4.01 x 10^-16^ | 8.02 x 10^-16^ | 1.20 (1.03-1.41) | | 0.022 | 0.022 | | 0.99 (0.84-1.16) | 0.855 | 0.855 | | 0.22 (-0.33-0.78) | 0.215 | 0.215 | 0.193 |
| PRS-SZ * Emotional abuse | 831 (475/356) | 2.34 (1.89-2.92) | 2.43 x 10^-14^ | 2.43 x 10^-14^ | 2.40 (1.97-2.97) | | 7.93 x 10^-17^ | 3.17 x 10^-16^ | | 0.97 (0.78-1.20) | 0.777 | 0.855 | | 1.70 (0.00-3.40) | 0.025 | 0.096 | 0.309 |
| PRS-SZ * Sexual abuse | 832 (476/356) | 2.34 (1.91-2.89) | 7.43 x 10^-16^ | 9.91 x 10^-16^ | 1.66 (1.35-2.10) | | 5.62 x 10^-6^ | 7.49 x 10^-6^ | | 0.89 (0.73-1.09) | 0.263 | 0.855 | | 0.47 (-0.45-1.39) | 0.161 | 0.214 | 0.233 |

All variables were adjusted for age, sex and education.

**Notes:** ETI-SR: Early Trauma Inventory-Self Report, FDR: False discovery rate, HCs: Healthy Controls, OR: Odd Ratio, PRS-SZ: Polygenic Risk Score-Schizophrenia, RERI: Relative Excess Risk due to Interaction, SSDs: Schizophrenia Spectrum Disorders.

Supplementary Table 7. Main and interaction effects of PRS-SZ and subdomains of the K-PERS-I on the PANSS or BCSS in patients with SSDs

| Clinical phenotypes | Models^*^ | N | PRS-SZ | | | | Environmental measure | | | | Additive Interaction | | | | R^2^ |
| --- | --- | --- | --- | --- | --- | --- | --- | --- | --- | --- | --- | --- | --- | --- | --- |
|  |  |  | Beta | SD | p-value | FDR  value | Beta | SD | p-value | FDR  value | Beta | SD | p-value | FDR  value |  |
| PANSS |  |  |  |  |  |  |  |  |  |  |  |  |  |  |  |
| Total | PRS-SZ * Paternal age at birth | 385 | 1.62 | 1.43 | 0.257 | 0.257 | 0.84 | 0.97 | 0.382 | 0.382 | -0.03 | 0.99 | 0.980 | 0.980 | 0.037 |
|  | PRS-SZ * Obstetric complications | 355 | 1.74 | 1.54 | 0.259 | 0.259 | 1.31 | 0.85 | 0.125 | 0.125 | -0.93 | 0.91 | 0.304 | 0.304 | 0.048 |
|  | PRS-SZ * Parental SES | 388 | 1.69 | 1.41 | 0.231 | 0.231 | -0.83 | 1.02 | 0.415 | 0.415 | -1.00 | 1.12 | 0.373 | 0.373 | 0.039 |
|  | PRS-SZ * Urbanicity | 390 | 1.72 | 1.42 | 0.225 | 0.225 | 1.03 | 1.07 | 0.336 | 0.336 | 0.67 | 1.14 | 0.559 | 0.559 | 0.038 |
|  | PRS-SZ * Childhood adversity | 382 | 1.55 | 1.56 | 0.321 | 0.321 | 0.24 | 1.18 | 0.836 | 0.836 | 0.36 | 1.29 | 0.781 | 0.781 | 0.037 |
|  | PRS-SZ * Recent life events | 330 | 1.51 | 1.59 | 0.342 | 0.342 | 0.27 | 1.09 | 0.801 | 0.801 | -1.00 | 1.23 | 0.417 | 0.417 | 0.057 |
| PANSS subdomains | |  |  |  |  |  |  |  |  |  |  |  |  |  |  |
| Positive  symptoms | PRS-SZ * Paternal age at birth | 388 | -0.01 | 0.50 | 0.986 | 0.986 | 0.21 | 0.33 | 0.535 | 0.582 | 0.08 | 0.35 | 0.818 | 0.907 | 0.034 |
|  | PRS-SZ * Obstetric complications | 358 | 0.26 | 0.53 | 0.626 | 0.626 | 0.54 | 0.29 | 0.066 | 0.198 | -0.55 | 0.31 | 0.078 | 0.234 | 0.062 |
|  | PRS-SZ * Parental SES | 391 | 0.05 | 0.49 | 0.925 | 0.925 | 0.08 | 0.35 | 0.814 | 0.814 | 0.06 | 0.39 | 0.879 | 0.879 | 0.031 |
|  | PRS-SZ * Urbanicity | 393 | 0.07 | 0.49 | 0.886 | 0.886 | 0.43 | 0.37 | 0.247 | 0.464 | -0.06 | 0.40 | 0.871 | 0.874 | 0.037 |
|  | PRS-SZ * Childhood adversity | 385 | 0.20 | 0.54 | 0.716 | 0.716 | 0.88 | 0.41 | 0.031 | 0.093 | 0.01 | 0.45 | 0.990 | 0.995 | 0.050 |
|  | PRS-SZ * Recent life events | 333 | -0.42 | 0.56 | 0.453 | 0.453 | -0.02 | 0.39 | 0.967 | 0.967 | 0.18 | 0.44 | 0.683 | 0.683 | 0.057 |
| Negative  symptoms | PRS-SZ * Paternal age at birth | 387 | 0.97 | 0.50 | 0.052 | 0.156 | 0.19 | 0.34 | 0.582 | 0.582 | -0.21 | 0.35 | 0.538 | 0.907 | 0.021 |
|  | PRS-SZ * Obstetric complications | 357 | 0.80 | 0.54 | 0.137 | 0.411 | 0.23 | 0.30 | 0.449 | 0.449 | -0.23 | 0.32 | 0.478 | 0.717 | 0.023 |
|  | PRS-SZ * Parental SES | 390 | 0.90 | 0.49 | 0.065 | 0.195 | -0.33 | 0.35 | 0.352 | 0.528 | -0.82 | 0.39 | 0.037 | 0.111 | 0.034 |
|  | PRS-SZ * Urbanicity | 392 | 0.95 | 0.49 | 0.055 | 0.165 | -0.02 | 0.37 | 0.962 | 0.962 | 0.50 | 0.40 | 0.210 | 0.630 | 0.027 |
|  | PRS-SZ * Childhood adversity | 384 | 0.81 | 0.54 | 0.130 | 0.390 | -0.76 | 0.41 | 0.062 | 0.093 | 0.00 | 0.44 | 0.995 | 0.995 | 0.032 |
|  | PRS-SZ * Recent life events | 332 | 1.16 | 0.55 | 0.036 | 0.108 | -0.18 | 0.38 | 0.638 | 0.957 | -0.81 | 0.43 | 0.059 | 0.177 | 0.037 |
| General  Psychopathology | PRS-SZ * Paternal age at birth | 387 | 0.62 | 0.68 | 0.360 | 0.540 | 0.31 | 0.46 | 0.495 | 0.582 | -0.06 | 0.47 | 0.907 | 0.907 | 0.052 |
|  | PRS-SZ * Obstetric complications | 357 | 0.61 | 0.74 | 0.410 | 0.615 | 0.55 | 0.41 | 0.175 | 0.263 | -0.15 | 0.44 | 0.736 | 0.736 | 0.057 |
|  | PRS-SZ * Parental SES | 390 | 0.67 | 0.67 | 0.319 | 0.479 | -0.45 | 0.48 | 0.350 | 0.528 | -0.17 | 0.53 | 0.744 | 0.879 | 0.055 |
|  | PRS-SZ * Urbanicity | 392 | 0.61 | 0.67 | 0.364 | 0.546 | 0.52 | 0.51 | 0.309 | 0.464 | 0.09 | 0.54 | 0.874 | 0.874 | 0.052 |
|  | PRS-SZ * Childhood adversity | 384 | 0.55 | 0.74 | 0.452 | 0.678 | 0.17 | 0.56 | 0.757 | 0.757 | 0.20 | 0.61 | 0.742 | 0.995 | 0.053 |
|  | PRS-SZ * Recent life events | 332 | 0.72 | 0.76 | 0.340 | 0.453 | 0.35 | 0.52 | 0.504 | 0.957 | -0.48 | 0.59 | 0.417 | 0.626 | 0.076 |
| BCSS |  |  |  |  |  |  |  |  |  |  |  |  |  |  |  |
| Negative-self | PRS-SZ * Paternal age at birth | 372 | 0.11 | 0.33 | 0.731 | 0.898 | -0.07 | 0.22 | 0.759 | 0.916 | 0.02 | 0.23 | 0.930 | 0.930 | 0.044 |
|  | PRS-SZ * Obstetric complications | 345 | 0.06 | 0.36 | 0.866 | 0.866 | 0.27 | 0.19 | 0.150 | 0.194 | 0.16 | 0.20 | 0.414 | 0.414 | 0.045 |
|  | PRS-SZ * Parental SES | 374 | 0.19 | 0.33 | 0.560 | 0.984 | -0.12 | 0.23 | 0.605 | 0.605 | -0.40 | 0.26 | 0.126 | 0.252 | 0.051 |
|  | PRS-SZ * Urbanicity | 375 | 0.11 | 0.33 | 0.741 | 0.903 | -0.04 | 0.24 | 0.860 | 0.860 | -0.03 | 0.27 | 0.919 | 0.919 | 0.045 |
|  | PRS-SZ * Childhood adversity | 368 | -0.05 | 0.36 | 0.901 | 0.901 | 0.72 | 0.27 | 0.008 | 0.008 | 0.52 | 0.30 | 0.081 | 0.162 | 0.076 |
|  | PRS-SZ * Recent life events | 314 | 0.13 | 0.39 | 0.747 | 0.803 | 0.48 | 0.26 | 0.067 | 0.134 | -0.11 | 0.30 | 0.705 | 0.705 | 0.055 |
| Negative-others | PRS-SZ * Paternal age at birth | 372 | 0.04 | 0.31 | 0.898 | 0.898 | 0.02 | 0.21 | 0.916 | 0.916 | -0.16 | 0.22 | 0.478 | 0.930 | 0.017 |
|  | PRS-SZ * Obstetric complications | 345 | -0.08 | 0.34 | 0.823 | 0.866 | 0.23 | 0.18 | 0.194 | 0.194 | 0.55 | 0.19 | 0.004 | 0.008 | 0.043 |
|  | PRS-SZ * Parental SES | 374 | 0.01 | 0.31 | 0.984 | 0.984 | 0.12 | 0.22 | 0.601 | 0.605 | 0.04 | 0.25 | 0.889 | 0.889 | 0.017 |
|  | PRS-SZ * Urbanicity | 375 | 0.04 | 0.31 | 0.903 | 0.903 | -0.06 | 0.23 | 0.812 | 0.86 | 0.34 | 0.25 | 0.171 | 0.342 | 0.022 |
|  | PRS-SZ * Childhood adversity | 368 | -0.05 | 0.34 | 0.889 | 0.901 | 1.04 | 0.25 | <0.001 | <0.001 | 0.29 | 0.28 | 0.311 | 0.311 | 0.065 |
|  | PRS-SZ * Recent life events | 314 | 0.09 | 0.36 | 0.803 | 0.803 | 0.16 | 0.25 | 0.524 | 0.524 | -0.31 | 0.28 | 0.265 | 0.530 | 0.017 |

^*^Only interaction term described; All variables were adjusted for age, sex and education.

**Notes**: BCSS: Brief Core Schema Scales, FDR: False discovery rate, K-PERS-I: Korea Polyenvironmental Risk Score-I, N: Number, PANSS: Positive and Negative Syndrome Scale, PRS-SZ: Polygenic Risk Score-Schizophrenia, SD: Standard Deviation, SES: Socio-Economic Status, SSDs: Schizophrenia Spectrum Disorders.

Supplementary Table 8. Main and interaction effects of PRS-SZ and subdomains of the ETI-SR on the PANSS or BCSS in patients with SSDs

| Phenotypes | Models^*^ | N | PRS-SZ | | | | Environmental measure | | | | Additive Interaction | | | | R^2^ |
| --- | --- | --- | --- | --- | --- | --- | --- | --- | --- | --- | --- | --- | --- | --- | --- |
|  |  |  | Beta | SD | p-value | FDR  value | Beta | SD | p-value | FDR  value | Beta | SD | p-value | FDR  value |  |
| PANSS |  |  |  |  |  |  |  |  |  |  |  |  |  |  |  |
| Total | PRS-SZ * General Trauma | 471 | 2.68 | 1.25 | 0.032 | 0.032 | 0.56 | 0.80 | 0.483 | 0.483 | -0.24 | 0.97 | 0.804 | 0.804 | 0.055 |
|  | PRS-SZ * Physical Punishment | 471 | 2.62 | 1.23 | 0.033 | 0.033 | 1.30 | 0.89 | 0.147 | 0.147 | -0.10 | 1.05 | 0.927 | 0.927 | 0.058 |
|  | PRS-SZ * Emotional abuse | 471 | 2.64 | 1.25 | 0.036 | 0.036 | 2.03 | 0.82 | 0.014 | 0.014 | 0.15 | 0.93 | 0.872 | 0.872 | 0.067 |
|  | PRS-SZ * Sexual abuse | 472 | 2.53 | 1.24 | 0.042 | 0.042 | 0.74 | 0.74 | 0.314 | 0.314 | 0.17 | 0.76 | 0.826 | 0.826 | 0.056 |
| PANSS subdomains | |  |  |  |  |  |  |  |  |  |  |  |  |  |  |
| Positive  symptoms | PRS-SZ * General Trauma | 475 | 0.45 | 0.43 | 0.304 | 0.304 | 0.68 | 0.28 | 0.015 | 0.045 | -0.25 | 0.34 | 0.463 | 0.964 | 0.042 |
|  | PRS-SZ * Physical Punishment | 475 | 0.39 | 0.43 | 0.365 | 0.365 | 0.79 | 0.31 | 0.011 | 0.033 | -0.23 | 0.37 | 0.537 | 0.827 | 0.043 |
|  | PRS-SZ * Emotional abuse | 475 | 0.42 | 0.44 | 0.339 | 0.339 | 0.99 | 0.29 | 5.71 x 10^-4^ | 0.002 | -0.10 | 0.33 | 0.764 | 0.764 | 0.054 |
|  | PRS-SZ * Sexual abuse | 476 | 0.40 | 0.43 | 0.361 | 0.361 | 0.40 | 0.26 | 0.119 | 0.357 | -0.19 | 0.26 | 0.474 | 0.711 | 0.035 |
| Negative  symptoms | PRS-SZ * General Trauma | 471 | 0.96 | 0.42 | 0.023 | 0.048 | -0.33 | 0.27 | 0.221 | 0.332 | -0.04 | 0.33 | 0.892 | 0.964 | 0.026 |
|  | PRS-SZ * Physical Punishment | 471 | 0.99 | 0.42 | 0.018 | 0.048 | -0.17 | 0.30 | 0.573 | 0.573 | -0.08 | 0.36 | 0.827 | 0.827 | 0.023 |
|  | PRS-SZ * Emotional abuse | 471 | 1.02 | 0.43 | 0.017 | 0.051 | 0.06 | 0.28 | 0.838 | 0.838 | -0.10 | 0.32 | 0.750 | 0.764 | 0.022 |
|  | PRS-SZ * Sexual abuse | 472 | 0.90 | 0.42 | 0.033 | 0.052 | -0.05 | 0.25 | 0.852 | 0.852 | 0.24 | 0.26 | 0.351 | 0.711 | 0.024 |
| General  Psychopathology | PRS-SZ * General Trauma | 473 | 1.32 | 0.61 | 0.032 | 0.048 | 0.20 | 0.40 | 0.608 | 0.608 | 0.02 | 0.48 | 0.964 | 0.964 | 0.078 |
|  | PRS-SZ * Physical Punishment | 473 | 1.30 | 0.61 | 0.032 | 0.048 | 0.63 | 0.44 | 0.156 | 0.234 | 0.17 | 0.52 | 0.745 | 0.827 | 0.082 |
|  | PRS-SZ * Emotional abuse | 473 | 1.26 | 0.62 | 0.041 | 0.061 | 0.97 | 0.41 | 0.017 | 0.026 | 0.32 | 0.46 | 0.486 | 0.764 | 0.092 |
|  | PRS-SZ * Sexual abuse | 474 | 1.29 | 0.61 | 0.035 | 0.052 | 0.36 | 0.36 | 0.312 | 0.468 | 0.09 | 0.37 | 0.808 | 0.808 | 0.080 |
| BCSS |  |  |  |  |  |  |  |  |  |  |  |  |  |  |  |
| Negative-self | PRS-SZ * General Trauma | 433 | 0.15 | 0.31 | 0.639 | 0.639 | 0.60 | 0.22 | 0.007 | 0.007 | 0.21 | 0.27 | 0.437 | 0.874 | 0.049 |
|  | PRS-SZ * Physical Punishment | 433 | 0.08 | 0.31 | 0.790 | 0.790 | 0.70 | 0.22 | 0.002 | 0.002 | 0.41 | 0.28 | 0.142 | 0.284 | 0.062 |
|  | PRS-SZ * Emotional abuse | 433 | 0.13 | 0.29 | 0.671 | 0.671 | 1.29 | 0.22 | 9.41 x 10^-9^ | 9.41 x 10^-9^ | 0.46 | 0.26 | 0.074 | 0.148 | 0.126 |
|  | PRS-SZ * Sexual abuse | 434 | 0.10 | 0.31 | 0.759 | 0.759 | 0.68 | 0.22 | 0.002 | 0.002 | 0.23 | 0.22 | 0.298 | 0.596 | 0.053 |
| Negative-others | PRS-SZ * General Trauma | 433 | 0.18 | 0.29 | 0.541 | 0.639 | 1.02 | 0.21 | 1.29 x 10^-6^ | 2.58 x 10^-6^ | -0.01 | 0.25 | 0.982 | 0.982 | 0.071 |
|  | PRS-SZ * Physical Punishment | 433 | 0.11 | 0.29 | 0.712 | 0.790 | 1.20 | 0.21 | 2.01 x 10^-8^ | 4.02 x 10^-8^ | 0.23 | 0.26 | 0.375 | 0.375 | 0.099 |
|  | PRS-SZ * Emotional abuse | 433 | 0.13 | 0.28 | 0.633 | 0.671 | 1.51 | 0.21 | 2.05 x 10^-12^ | 4.10 x 10^-12^ | 0.18 | 0.24 | 0.470 | 0.47 | 0.139 |
|  | PRS-SZ * Sexual abuse | 434 | 0.11 | 0.29 | 0.699 | 0.759 | 0.84 | 0.21 | 5.31 x 10^-5^ | 1.06 x 10^-4^ | 0.02 | 0.21 | 0.917 | 0.917 | 0.052 |

^*^Only interaction term described; All variables were adjusted for age, sex and education.

**Notes**: BCSS: Brief Core Schema Scales, ETI-SR: Early Trauma Inventory-Self Report, FDR: False discovery rate, N: Number, PANSS: Positive and Negative Syndrome Scale, PRS-SZ: Polygenic Risk Score-Schizophrenia, SD: Standard Deviation, SSDs: Schizophrenia Spectrum Disorders.

Supplementary Table 9. Comparing the sample sizes of raw data and imputed data in patients with SSDs and HCs

|  | Raw data | | | Imputed data | | |
| --- | --- | --- | --- | --- | --- | --- |
|  | Total | SSDs | HCs | Total | SSDs | HCs |
| K-PERS-I | 615 | 295 | 320 | 752 | 397 | 355 |
| PANSS |  | 288 |  |  | 389 |  |
| BCSS |  | 280 |  |  | 375 |  |
| ETI-SR | 830 | 474 | 356 | 832 | 476 | 356 |
| PANSS |  | 470 |  |  | 472 |  |
| BCSS |  | 432 |  |  | 434 |  |

**Notes:** BCSS: Brief Core Schema Scales, ETI-SR: Early Trauma Inventory Self Report, HCs: Healthy Controls, K-PERS-I: Korea Polyenvironmental Risk Score-I, PANSS: Positive and Negative Syndrome Scale, SSDs: Schizophrenia Spectrum Disorder

Supplementary Table 10. Main and interaction effects of PRS-SZ and K-PERS-I/ETI-SR on case-control status after imputation

| Models | Total (SSDs/HCs) | PRS-SZ | | Environmental measures | | Multiplicative Interaction | | Nagelkerke’s R^2^ |
| --- | --- | --- | --- | --- | --- | --- | --- | --- |
|  |  | Adjusted OR (95% CI) | p-value | Adjusted OR (95% CI) | p-value | Adjusted OR (95% CI) | p-value |  |
| PRS-SZ | 752 (397/355) | 2.23 (1.81-2.76) | 9.31E-14 | - | - | - | - | 0.163 |
| K-PERS-I |  | - | - | 3.14 (2.61-3.81) | 5.99E-33 | - | - | 0.334 |
| PRS-SZ + K-PERS-I |  | 2.43 (1.91-3.13) | 1.75E-12 | 3.26 (2.68-3.99) | 1.12E-31 | - | - | 0.405 |
| PRS-SZ + K-PERS-I + PRS-SZ * K-PERS-I |  | 2.43 (1.9-3.13) | 2.40E-12 | 3.21 (2.64-3.95) | 3.03E-30 | 0.93 (0.75-1.15) | 0.505 | 0.406 |
| PRS-SZ | 832 (476/356) | 2.30 (1.89-2.82) | 4.44E-16 | - | - | - | - | 0.186 |
| ETI-SR |  | - | - | 1.93 (1.63-2.31) | 1.37E-13 | - | - | 0.173 |
| PRS-SZ + ETI-SR |  | 2.39 (1.95-2.97) | 4.86E-16 | 1.99 (1.66-2.40) | 1.54E-13 | - | - | 0.272 |
| PRS-SZ + ETI-SR + PRS-SZ * ETI-SR |  | 2.39 (1.94-2.96) | 6.93E-16 | 1.97 (1.64-2.39) | 1.04E-12 | 0.96 (0.79-1.17) | 0.697 | 0.272 |

All variables were adjusted for age, sex and education.

**Notes**: ETI-SR: Early Trauma Inventory Self Report, HCs: Healthy Controls, K-PERS-I: Korea Polyenvironmental Risk Score-I, OR: Odd Ratio, PRS-SZ: Polygenic Risk Score-Schizophrenia, SSDs: Schizophrenia Spectrum Disorders

Supplementary Table 11. Main and interaction effects of PRS-SZ and K-PERS-I/ETI-SR on the PANSS or BCSS in patients with SSDs after imputation

| Phenotypes | Models | N | PRS-SZ | | | Environmental measures | | | Additive Interaction | | | R^2^ |
| --- | --- | --- | --- | --- | --- | --- | --- | --- | --- | --- | --- | --- |
|  |  |  | Beta | SD | p-value | Beta | SD | p-value | Beta | SD | p-value |  |
| PANSS |  |  |  |  |  |  |  |  |  |  |  |  |
| Total | PRS-SZ + K-PERS-I + PRS-SZ * K-PERS-I | 389 | 2.09 | 1.55 | 0.177 | 1.09 | 1.13 | 0.333 | -0.83 | 1.26 | 0.509 | 0.038 |
|  | PRS-SZ + ETI-SR + PRS-SZ * ETI-SR | 472 | 2.64 | 1.26 | 0.036 | 1.50 | 0.80 | 0.061 | 0.17 | 0.92 | 0.853 | 0.061 |
| Positive  symptoms | PRS-SZ + K-PERS-I + PRS-SZ * K-PERS-I | 392 | 0.17 | 0.54 | 0.755 | 0.67 | 0.39 | 0.089 | -0.07 | 0.44 | 0.865 | 0.041 |
|  | PRS-SZ + ETI-SR + PRS-SZ * ETI-SR | 476 | 0.46 | 0.44 | 0.291 | 0.93 | 0.28 | 0.001 | -0.15 | 0.32 | 0.641 | 0.052 |
| Negative  symptoms | PRS-SZ + K-PERS-I + PRS-SZ * K-PERS-I | 391 | 1.13 | 0.54 | 0.036 | -0.38 | 0.39 | 0.338 | -0.62 | 0.44 | 0.157 | 0.029 |
|  | PRS-SZ + ETI-SR + PRS-SZ * ETI-SR | 472 | 0.96 | 0.43 | 0.025 | -0.18 | 0.27 | 0.513 | 0.00 | 0.31 | 0.993 | 0.023 |
| General  Psychopathology | PRS-SZ + K-PERS-I + PRS-SZ * K-PERS-I | 391 | 0.80 | 0.73 | 0.274 | 0.73 | 0.54 | 0.177 | -0.31 | 0.60 | 0.602 | 0.056 |
|  | PRS-SZ + ETI-SR + PRS-SZ * ETI-SR | 474 | 1.28 | 0.62 | 0.039 | 0.71 | 0.39 | 0.069 | 0.28 | 0.45 | 0.535 | 0.085 |
| BCSS |  |  |  |  |  |  |  |  |  |  |  |  |
| Negative-self | PRS-SZ + K-PERS-I + PRS-SZ * K-PERS-I | 375 | 0.13 | 0.36 | 0.710 | 0.58 | 0.26 | 0.027 | 0.09 | 0.29 | 0.768 | 0.059 |
|  | PRS-SZ + ETI-SR + PRS-SZ * ETI-SR | 434 | 0.01 | 0.31 | 0.962 | 0.99 | 0.19 | 4.78.E-07 | 0.48 | 0.23 | 0.035 | 0.102 |
| Negative-others | PRS-SZ + K-PERS-I + PRS-SZ * K-PERS-I | 375 | -0.06 | 0.34 | 0.861 | 0.58 | 0.25 | 0.019 | 0.23 | 0.28 | 0.412 | 0.036 |
|  | PRS-SZ + ETI-SR + PRS-SZ * ETI-SR | 434 | 0.11 | 0.29 | 0.704 | 1.38 | 0.18 | 1.30.E-13 | 0.26 | 0.21 | 0.220 | 0.146 |

All variables were adjusted for age, sex and education.

**Notes:** BCSS: Brief Core Schema Scales, ETI-SR: Early Trauma Inventory Self Report, K-PERS-I: Korea Polyenvironmental Risk Score-I, N: Number of patients with SSDs, PANSS: Positive and Negative Syndrome Scale, PRS-SZ: Polygenic Risk Score-Schizophrenia, SD: Standard Deviation, SSDs: Schizophrenia Spectrum Disorder

**3. Power Calculation via Simulation in R (multiplication interaction)**

# Load necessary libraries

set.seed(123) # For reproducibility

# Define parameters for simulation

n_simulations <- 1000 # Number of simulations

n_obs <- 615 # Number of observations per simulation

n_predictors <- 3 # Number of additional covariates

beta_PRS <- log(2.43) # Log-odds (effect size) for X1

beta_Env <- log(3.37) # Log-odds (effect size) for X2

beta_interaction <- log(0.98) # Log-odds (effect size) for interaction term

prevalence <- 0.4796748 # Desired prevalence of the outcome

intercept <- log(prevalence / (1 - prevalence)) # Intercept to achieve the desired prevalence

alpha <- 0.05 # Significance level

# Function to calculate power for interaction term with main effects, interaction, and prevalence

calculate_power_interaction_with_prevalence <- function(n_obs) {

sig_count <- 0 # To count significant results for the interaction term

for (i in 1:n_simulations) {

# Simulate continuous predictors

PRS <- rnorm(n_obs, mean = 0, sd = 1) # Simulate standardised polygenic score

Env <- rnorm(n_obs, mean = 0, sd = 1) # Simulate standardised environmental score

interaction_term <- PRS * Env # Interaction between standardized PRS and Env

# Simulate additional covariates (uncorrelated for simplicity)

X_covariates <- matrix(rnorm(n_obs * n_predictors), ncol = n_predictors)

# Simulate log-odds including main effects, interaction effect, and baseline intercept for prevalence

log_odds <- intercept + beta_PRS * PRS + beta_Env * Env + beta_interaction * interaction_term + rnorm(n_obs) # Main and interaction effects

prob <- 1 / (1 + exp(-log_odds)) # Convert log-odds to probability

# Simulate binary outcome based on probabilities

Y <- rbinom(n_obs, 1, prob)

# Fit the logistic regression model with main effects, interaction, and covariates

model <- glm(Y ~ PRS * Env + X_covariates, family = binomial)

# Check if the interaction term is significant

p_value <- summary(model)$coefficients["PRS:Env", 4] # p-value of the interaction term

if (p_value < alpha) {

sig_count <- sig_count + 1 # Count if the interaction term is significant

}

}

# Estimate power for the interaction term

power_estimate <- sig_count / n_simulations

return(power_estimate)

}

# Calculate power for a specific sample size

power_estimate <- calculate_power_interaction_with_prevalence(n_obs)

cat("Estimated power for interaction term:", power_estimate, "\n")

### Iterative approach to estimate the sample size needed to achieve 80% power

# Iteratively increase sample size to achieve 80% power for interaction term (with prevalence)

set.seed(123) # For reproducibility

n_obs <- 175000 # Starting sample size

step_size <- 1000 # Increase sample size in steps

desired_power <- 0.8 # Target power

max_iterations <- 100 # Max number of iterations to avoid infinite loop

iteration <- 0

power_estimate <- 0

while (power_estimate < desired_power && iteration < max_iterations) {

power_estimate <- calculate_power_interaction_with_prevalence(n_obs)

cat("Sample size:", n_obs, "Estimated power for interaction:", power_estimate, "\n")

# Increase sample size if power is below the desired threshold

if (power_estimate < desired_power) {

n_obs <- n_obs + step_size

iteration <- iteration + 1

}

}

# Output final sample size

if (power_estimate >= desired_power) {

cat("Estimated sample size needed for 80% power for interaction term (with prevalence):", n_obs, "\n")

} else {

cat("Could not achieve 80% power after", max_iterations, "iterations.\n")

}

**4. Power Calculation via Simulation in R (additive interaction)**

### calculate RERI (ICR) -> delta method

##### Please cite as follows:

##### Mathur MB & VanderWeele TJ (2018). R function for additive interaction measures. Epidemiology 29(1), e5-e6.

##### Fn: Checks whether both exposure ORs are > 1

causative = function( OR10, OR01 ) {

return( (OR10 > 1) & (OR01 > 1) )

}

##### Fn: Compute additive interaction measures with inference

additive_interactions = function( model, dat=NULL, monotone=0, CI.level=0.95, recode=FALSE ) {

# check for valid logistic regression model

if ( is.null(model$family$link) ) {

stop ("Argument 'model' must be a logistic regression fit with glm()")

} else if ( !model$family$link == "logit" ) {

stop ("Argument 'model' must be a logistic regression fit with glm()")

}

# extract exposure variable names

exposure1 = names(coef(model))[5]

exposure2 = names(coef(model))[6]

# variables to keep for delta method

# requires that the two exposures of interest be listed first in model

# in order to get the right variables in the interaction string

interaction.string = paste(exposure1, exposure2, sep=":")

if ( !(interaction.string %in% names( coef(model) ) ) ) {

stop("Could not identify the interaction coefficient in the model formula, possibly because you used the I() operator. Please enter the interaction using * instead.")

}

keepers = c(exposure1, exposure2, interaction.string)

# get variance-covariance matrix of the GLM

V = as.matrix( vcov(model) )

V2 = V[keepers, keepers] # subset to just the coefficients of interest

# calculate various coefficients and ORs

b10 = coef(model)[exposure1]

b01 = coef(model)[exposure2]

bint = coef(model)[interaction.string]

OR00 = 1.0 # reference

OR10 = exp(b10)

OR01 = exp(b01)

OR11 = exp( b10 + b01 + bint )

# check if we are already in causative case

if( !causative( OR10 = OR10, OR01 = OR01 ) ) {

if(!recode) stop("Error: At least one exposure has negative association with outcome. Set argument recode=TRUE if you would like the exposures to be automatically recoded.")

if(recode) {

# attempt recoding

if (is.null(dat)) stop("Error: Provide data in order to recode preventive exposures")

# find stratum with lowest overall risk, conditional on covariates

temp = data.frame( cat = c("OR10", "OR01", "OR11"), value = c(OR10, OR01, OR11) )

refcat = temp$cat[ which.min(temp$value) ] # get category's name

# extract first "subscript" number

E1.ref = substr( refcat, 3, 3 )

# extract second "subscript" number

E2.ref = substr( refcat, 4, 4 )

# recode each exposure based on new reference category

dat[[exposure1]] = ifelse( dat[[exposure1]] == E1.ref, 0, 1 )

dat[[exposure2]] = ifelse( dat[[exposure2]] == E2.ref, 0, 1 )

# tell user what happened

warning("Recoding exposures; new reference category for ", exposure1, " is ", E1.ref,

" and for ", exposure2, " is ", E2.ref)

# refit model by refitting user's original call

# but with newly recoded dataframe

model = update(model, .~., data=dat)

# get new coefficients and ORs

b10 = coef(model)[exposure1]

b01 = coef(model)[exposure2]

bint = coef(model)[interaction.string]

OR00 = 1.0 # reference

OR10 = exp(b10)

OR01 = exp(b01)

OR11 = exp( b10 + b01 + bint )

}

}

# RERI (VanderWeele pg. 258-259)

RERI = OR11 - exp(b10) - exp(b01) + 1

require(msm)

SE.RERI = deltamethod( ~ exp( x1 + x2 + x3 ) - exp(x1) - exp(x2) + 1,

mean=c( coef(model)[exposure1], coef(model)[exposure2], coef(model)[interaction.string] ),

cov=V2) # this is its SE

# attributable proportion (VanderWeele pg. 256)

AP = RERI / OR11

SE.AP = deltamethod( ~ ( exp( x1 + x2 + x3 ) - exp(x1) - exp(x2) + 1 ) / exp( x1 + x2 + x3 ),

mean=c( coef(model)[exposure1], coef(model)[exposure2], coef(model)[interaction.string] ),

cov=V2) # this is its SE

alpha = 1-CI.level

z = qnorm( 1 - alpha/2 ) # critical value

# RERI p-values for tests vs. nulls of 0, 1, or 2

# for mechanistic conclusions (VanderWeele pg. 275)

p.0 = 1 - pnorm( RERI/SE.RERI )

p.1 = 1 - pnorm( (RERI-1) / SE.RERI )

p.2 = 1 - pnorm( (RERI-2) / SE.RERI )

# based on monotonicity assumption, give p-value for epistatic and sufficient-cause interactions

if (monotone==0) {

p.val.epi = p.2

p.val.suff.cause = p.1

} else if (monotone==1) {

p.val.epi = p.val.suff.cause = p.1

} else if (monotone==2) {

p.val.epi = p.val.suff.cause = p.0

} else {

stop("Argument 'monotone' must be 0, 1, or 2")

}

##### Prop. of joint effect due to each exposure separately or to their interaction ####

# VanderWeele pg. 282

denom = OR11 - 1

E1.contrib = ( exp( coef(model)[exposure1] ) - 1 ) / denom

E2.contrib = ( exp( coef(model)[exposure2] ) - 1 ) / denom

int.contrib = RERI / denom

SE.1 = deltamethod( ~ ( exp(x1) - 1 ) / ( exp( x1 + x2 + x3 ) - 1 ),

mean=c( coef(model)[exposure1], coef(model)[exposure2], coef(model)[interaction.string] ),

cov=V2)

SE.2 = deltamethod( ~ ( exp(x2) - 1 ) / ( exp( x1 + x2 + x3 ) - 1 ),

mean=c( coef(model)[exposure1], coef(model)[exposure2], coef(model)[interaction.string] ),

cov=V2)

SE.3 = deltamethod( ~ ( exp( x1 + x2 + x3 ) - exp(x1) - exp(x2) + 1 ) / ( exp( x1 + x2 + x3 ) - 1 ),

mean=c( coef(model)[exposure1], coef(model)[exposure2], coef(model)[interaction.string] ),

cov=V2)

alpha = 1-CI.level

z = qnorm( 1 - alpha/2 ) # critical value

##### Return results #####

rs = data.frame( Stat = c( "RERI", "AP", exposure1, exposure2, interaction.string ),

Est = c( RERI, AP, E1.contrib, E2.contrib, int.contrib ),

CI.lo = c( RERI - z*SE.RERI, AP - z*SE.AP, E1.contrib - z*SE.1, E2.contrib - z*SE.2, int.contrib - z*SE.3 ),

CI.hi = c( RERI + z*SE.RERI, AP + z*SE.AP, E1.contrib + z*SE.1, E2.contrib + z*SE.2, int.contrib + z*SE.3 ),

p.val.0 = c( p.0, ( 1 - pnorm( abs(AP)/SE.AP) ), ( 1 - pnorm(E1.contrib/SE.1) ), ( 1 - pnorm(E2.contrib/SE.2) ), ( 1 - pnorm(int.contrib/SE.3) ) ),

p.val.epi = c( p.val.epi, NA, NA, NA, NA ),

p.val.suff.cause = c( p.val.suff.cause, NA, NA, NA, NA )

)

rownames(rs) = NULL

if (monotone == 1) cat("\nAssuming AT LEAST ONE of exposures has positive monotonic effect\n\n")

if (monotone == 2) cat("\nAssuming BOTH exposures have positive monotonic effect\n\n")

print(rs)

invisible(rs)

}

# install.packages("msm")

library(msm)

###############################################################################

# Statistical power analysis

# Load necessary libraries

set.seed(123) # For reproducibility

# Define parameters for simulation

n_simulations <- 1000 # Number of simulations

n_obs <- 615 # Number of observations per simulation

n_predictors <- 3 # Number of additional covariates

beta_PRS <- log(2.43) # Log-odds (effect size) for X1

beta_Env <- log(3.37) # Log-odds (effect size) for X2

beta_interaction <- log(0.98) # Log-odds (effect size) for interaction term

prevalence <- 0.4796748 # Desired prevalence of the outcome

intercept <- log(prevalence / (1 - prevalence)) # Intercept to achieve the desired prevalence

alpha <- 0.05 # Significance level

# Function to calculate power for interaction term with main effects, interaction, and prevalence

calculate_power_interaction_with_prevalence <- function(n_obs) {

sig_count <- 0 # To count significant results for the interaction term

for (i in 1:n_simulations) {

# Simulate continuous predictors

PRS <- rnorm(n_obs, mean = 0, sd = 1) # Simulate standardised polygenic score

Env <- rnorm(n_obs, mean = 0, sd = 1) # Simulate standardised environmental score

interaction_term <- PRS * Env # Interaction between standardized PRS and Env

# Simulate additional covariates (uncorrelated for simplicity)

X_covariates <- matrix(rnorm(n_obs * n_predictors), ncol = n_predictors)

# Simulate log-odds including main effects, interaction effect, and baseline intercept for prevalence

log_odds <- intercept + beta_PRS * PRS + beta_Env * Env + beta_interaction * interaction_term + rnorm(n_obs) # Main and interaction effects

prob <- 1 / (1 + exp(-log_odds)) # Convert log-odds to probability

# Simulate binary outcome based on probabilities

Y <- rbinom(n_obs, 1, prob)

# Fit the logistic regression model with main effects, interaction, and covariates

model <- additive_interactions(glm(Y ~ X_covariates + PRS * Env, family = binomial))

# Check if the interaction term is significant

p_value <- model[1,5] # p-value of the interaction term

if (p_value < alpha) {

sig_count <- sig_count + 1 # Count if the interaction term is significant

}

}

# Estimate power for the interaction term

power_estimate <- sig_count / n_simulations

return(power_estimate)

}

# Calculate power for a specific sample size

power_estimate <- calculate_power_interaction_with_prevalence(n_obs)

cat("Estimated power for interaction term:", power_estimate, "\n")

### Iterative approach to estimate the sample size needed to achieve 80% power

# Iteratively increase sample size to achieve 80% power for interaction term (with prevalence)

set.seed(123) # For reproducibility

n_obs <- 500 # Starting sample size

step_size <- 10 # Increase sample size in steps

desired_power <- 0.8 # Target power

max_iterations <- 100 # Max number of iterations to avoid infinite loop

iteration <- 0

power_estimate <- 0

while (power_estimate < desired_power && iteration < max_iterations) {

power_estimate <- calculate_power_interaction_with_prevalence(n_obs)

cat("Sample size:", n_obs, "Estimated power for interaction:", power_estimate, "\n")

# Increase sample size if power is below the desired threshold

if (power_estimate < desired_power) {

n_obs <- n_obs + step_size

iteration <- iteration + 1

}

}

# Output final sample size

if (power_estimate >= desired_power) {

cat("Estimated sample size needed for 80% power for interaction term (with prevalence):", n_obs, "\n")

} else {

cat("Could not achieve 80% power after", max_iterations, "iterations.\n")

}
